# A short period of early life oxytocin treatment rescues social behavior dysfunction via suppression of hippocampal hyperactivity in male mice

**DOI:** 10.1038/s41380-022-01692-7

**Published:** 2022-07-15

**Authors:** Libiao Pan, Lu Zheng, Xiaotong Wu, Zhenggang Zhu, Siyu Wang, Yi Lu, Yang He, Qian Yang, Xiaolin Ma, Xiaomeng Wang, Hongbin Yang, Li Zhan, Yujian Luo, Xiangyao Li, Yudong Zhou, Xiaodong Wang, Jianhong Luo, Lang Wang, Shumin Duan, Hao Wang

**Affiliations:** 1 Department of Neurobiology and Department of Neurosurgery of Second Affiliated Hospital, Key Laboratory for Biomedical Engineering of Education Ministry, Zhejiang University School of Medicine, Hangzhou, Zhejiang 310058 China; 2 NHC and CAMS Key Laboratory of Medical Neurobiology, MOE Frontier Science Center for Brain Research and Brain Machine Integration, School of Brain Science and Brain Medicine, Zhejiang University, Hangzhou, Zhejiang 310058 China; 3 Department of Neurology of the First Affiliated Hospital, Interdisciplinary Institute of Neuroscience and Technology, Zhejiang University School of Medicine, Hangzhou, 310027 China

**Keywords:** Neuroscience, Autism spectrum disorders

## Abstract

Early sensory experiences interact with genes to shape precise neural circuits during development. This process is vital for proper brain function in adulthood. Neurological dysfunctions caused by environmental alterations and/or genetic mutation may share the same molecular or cellular mechanisms. Here, we show that early life bilateral whisker trimming (BWT) subsequently affects social discrimination in adult male mice. Enhanced activation of the hippocampal dorsal CA3 (dCA3) in BWT mice was observed during social preference tests. Optogenetic activation of dCA3 in naive mice impaired social discrimination, whereas chemogenetic silencing of dCA3 rescued social discrimination deficit in BWT mice. Hippocampal oxytocin (OXT) is reduced after whisker trimming. Neonatal intraventricular compensation of OXT relieved dCA3 over-activation and prevented social dysfunction. Neonatal knockdown of OXT receptor in dCA3 mimics the effects of BWT, and cannot be rescued by OXT treatment. Social behavior deficits in a fragile X syndrome mouse model (Fmr1 KO mice) could also be recovered by early life OXT treatment, through negating dCA3 over-activation. Here, a possible avenue to prevent social dysfunction is uncovered.

## Introduction

Neurons in the adult brain make precise connections between each other to form the appropriate neural circuits required for proper brain function [1, 2]. During early postnatal development, such neural connectivity is first established by genetic programs and intrinsic activity [3]. Beyond that stage, the remarkably dynamic neonate brain undergoes a massive amount of wiring and re-wiring in response to the changing environment in which the individual is immersed [4, 5]. Information from the somatosensory system [6, 7], visual system [8, 9], and other sensory modalities [10, 11] all play critical roles in the process of shaping this neuronal connectivity. Among these, the somatosensory system is particularly important as early tactile experiences also serve as fundamental contributors to the correct establishment of social communication, object recognition, and motor control in many mammalian species including humans and rodents [12–14]. For example, depending on the precise manipulation timing, early life whisker deprivation in rodents can result in long-standing effects upon synaptic function and brain architecture within the somatosensory system potentially leading to altered behavioral outputs including those related to tactile discrimination and/or social behavior in adulthood [15–17]. Interestingly, many of the alterations caused by whisker trimming mimic the symptoms of human neuronal developmental disorders, particularly in patients with autism spectrum disorders (ASD), and show similar phenotypes to mouse models for ASD such as social behavior dysfunctions and tactile processing difficulties. Specifically, in the mouse model for fragile X syndrome (FXS), a disorder caused by a mutation of the gene that codes for the FMR1 protein, profound deficits are observed in social interaction and in the organization of the somatosensory cortex [18]. Therefore, it is possible that behavioral abnormalities caused by environmental alterations or genetic mutations share the same molecular or cellular mechanisms. As yet, such key links have not been fully addressed.

The social hormone oxytocin (OXT), a nine amino acid–long cyclical peptide that is synthesized in the paraventricular nucleus (PVN), supraoptic nucleus (SON), and accessory magnocellular hypothalamic nuclei, might be a potential candidate to connect the phenotypes caused by sensory experience deprivation and genetic mutations [19–21]. PVN OXT neurons extensively innervate to many emotion- and social-related nuclei, including the ventral tegmentum area (VTA), nucleus accumbens (NAc), amygdala, hippocampus and prefrontal cortex, etc., linking to functions such as participation in social recognition memory, mother-infant bonding, maternal nurturing, and pair-bonding [22]. Although those neurons do not receive direct inputs from the somatosensory system, a proportion of PVN OXT neurons respond to gentle touch [23], presumably through polysynaptic transmissions. In addition, OXT levels in the brain and the number of OXT-positive cells in the PVN can be significantly lower than normal in mice that have undergone early life whisker trimming [19]. These studies indicate that tactile experiences have a profound impact on oxytonergic signaling. The reduction of plasmatic and cerebral OXT levels is detected in both human patients with autism and mouse models for ASD [24, 25]. Exogenous acute OXT treatment in juvenile or adult improves the performance of social interaction of autism patients with low serum OXT level [25, 26], and restores impaired social recognition memory and social interactions in different ASD rodent models including Shank3-deficient rats and Shank3b-knockout mice [27, 28]. Recent studies indicate that OXT treatment in infancy improves long-lasting benefits for social behaviors until adulthood in Magel2- and Cntnap2-knockout mice [20, 21, 29]. In this, although the hippocampus might be the key, the exact underlying mechanisms of how OXT treatment ameliorates such social-behavioral dysfunction are not well understood.

Over the past decades, there has been an increasing focus on research relating to the exploration of the neural circuit basis of social behavior. These studies have highlighted the important roles of the hippocampus and VTAs in distinct aspects of social behavior. For example, the recruitment of hippocampal ventral CA1 and CA2 is essential for social memory [30, 31], whereas the activity dynamics of the VTA-to-NAc projection encodes and predicts key features of social interaction [32]. Furthermore, PVN OXT cells directly innervate to principle cells of the hippocampal CA2 area and can modulate the CA2 firing mode and alter social information processing [33]. OXT receptor signaling in the hippocampus is also recognized as required for some components of social recognition [34]. Although these prior studies lay a foundation for the essential role of oxytonergic signaling in the hippocampus and other brain areas in the regulation of social behavior, little is known relating to what underlies related pathological conditions. In this study, we aim to explore the relationship and key links between social-behavioral dysfunction caused by early life whisker deprivation and that caused by a genetic mutation. A better understanding of this question from the perspective focusing on the common core phenotypes may offer new insights and hold great promise for developing early intervention approaches to related neuronal developmental diseases such as autism.

## Material and methods

### Subjects

All experimental procedures were conducted according to the guidelines of Zhejiang University Animal Experimentation Committee. C57BL/6J male mice (RRID:IMSR_JAX:000664) and Fmr1^tm1Cgr^ transgenic male mice (RRID:MGI:5658228) were used. Mice were randomly assigned to either control or experimental groups. Prior to the experimental period the animals were housed in the chambers within custom-designed stainless-steel cabinets, under a constant temperature (22–23 °C), humidity (40–60%), and circadian cycle (12 h light/dark cycles, starting at 07:00). Food and water were available ad libitum.

### Whisker trimming

C57BL/6J mice pups, aged between P12 and P16 were used. For whisker trimming, P12 pups were anesthetized using isoflurane, and then their large whiskers were gently trimmed by using a pair of forceps under a stereoscope. The whiskers were checked every day and trimmed again, if and when necessary, to make sure no whisker sensory experience was attained between P12 and P16. Littermates were used as control mice that underwent the same procedure including anesthetic, but without any whisker trimming experience.

### Viral injection and stereotaxic surgeries

AAV-CaMKIIα-mCherry (AAV2/9, 1.00 × 10^12^ genomic copies per ml) and AAV-CaMKIIα-hChR2(H134R)-mCherry (AAV2/9, 7.57 × 10^12^ genomic copies per ml) were made by Shanghai SunBio Biomedical Technology Co., Ltd. (Shanghai, China). AAV-CaMKIIα-hM4Di-eGFP (AAV2/9, 2.96 × 10^13^ genomic copies per ml), AAV-CaMKIIα-eGFP (AAV2/9, 1.91 × 10^13^ genomic copies per ml) were made by Shanghai Taitool Bioscience Co., Ltd. (Shanghai, China). Before stereotaxic injection, mice were anesthetized with sodium pentobarbital (1% wt/vol) and then a volume of 50–100 nl (depending on viral titer and expression strength) virus solution was injected at the proper location. For optogenetic surgeries, stereotaxic injection of AAV-CaMKIIα-mCherry or AAV-CaMKIIα-hChR2(H134R)-mCherry was given into left-lateral CA3 (AP, −1.70 mm; ML, 2.00 mm; DV, −2.05 mm) in C57BL/6J male wild-type mice. After the injection, an implantable optic fiber (200 μm core diameter; 6 mm long; Newton, Hangzhou) was implanted above the injection site (dCA3: AP, −1.70 mm; ML, 2.00 mm; DV, −1.85 mm). For pharmacogenetic experiments, adult bilateral whisker trimming (BWT) (early life BWT) and Fmr1 KO mice were bilaterally injected in the dorsal CA3 (AP, −1.70 mm; ML, ±2.00 mm; DV, −2.05 mm) with AAV-CaMKIIα-hM4Di-EGFP or AAV-CaMKIIα-EGFP. We injected the virus into each location at 0.01 µl min^−1^. The syringe was not removed until 15–20 min after the end of infusion to allow time for the diffusion of the virus. After surgeries, the mice were returned to their home cages for 3–4 weeks for recovery and viral expression. For RNAi experiments, Oxtr siRNA targeting 330–348 nt (5’-GCTGTGTCGTCTGGTCAAA-3’) was designed as reported previously [19]. Sequences encoding shRNA were inserted into a pLentai-hU6-MCS-hEF1a-EGFP-3xFlag vector. The RNAi construct was confirmed by DNA sequencing. A lentivirus expressing Oxtr siRNA (3.63 × 10^9^ genomic copies per ml, 250 nl) or control siRNA (1.51 × 10^9^ genomic copies per ml, 250 nl) made by Shanghai Taitool Bioscience Co., Ltd. (Shanghai, China) were bilaterally injected into the dorsal CA3 (1.78 mm from posterior fontanelle; ML, ±1.78 mm; DV, −2.00 mm) in C57BL/6J mice at P7. The efficiency of the Oxtr siRNA construct was tested at P12 with western blot analysis using the tissue samples collected from hippocampal dorsal CA3.

### In vivo optogenetic stimulation

The C57BL/6J mice which underwent virus injection and optical fiber implant surgeries were used for all behavior tests involving optical stimulation. All test mice were habituated to the fiber connection for at least 2 days before testing. On the experiment day, the cannula was connected via an optic fiber sleeve to a 473 nm laser. In all behavior tests, the power of the laser was 0.5–2 mW with a repetition rate of 10 Hz and a laser pulse width of 5 ms. Laser stimulation was delivered 30 s per min. Any mice with missed injections or cannula locations were excluded. The mCherry-expressed mice underwent the same procedure and received the same intensity of laser stimulation.

### Slice preparation

Sagittal slices were obtained using the methods described previously [35]. In brief, mice were anesthetized with sodium pentobarbital, and then brain tissue was collected and perfused with an ice-cold oxygenated slicing solution. After decapitation, brains were removed rapidly for sectioning in an ice-cold slicing solution containing (in mM) 110 choline chloride, 7 MgCl_2_·6H_2_O, 2.5 KCl, 0.5 CaCl_2_·H_2_O, 1.3 NaH_2_PO_4_, 25 NaHCO_3_, 20 glucose, saturated with 95% O_2_ and 5% CO_2_. Coronal slices (300 μm) were prepared using a vibratome (Leica VT1000). Slices were recovered for 1 h at physiological temperature and then transferred to a recording chamber for recording in artificial cerebrospinal fluid (ACSF) containing (in mM): 125 NaCl, 2.5 KCl, 2 CaCl_2_·H_2_O, 1.3 MgCl_2_·6H_2_O, 1.3 NaH_2_PO_4_, 25 NaHCO_3_, and 10 glucose.

### Patch-clamp recording

Recordings were undertaken in 2–3 months or 17 days old C57BL/6J mice and Fmr1 KO mice at room temperature. Electrodes had resistances between 3 and 5 MΩ. Whole-cell voltage-clamp recordings were acquired from the soma of dorsal CA3 neurons using an Axopatch 200B amplifier and Digidata 1322A with pCLAMP 8.1 software (Molecular Devices). Signals were filtered at 2 kHz and digitized at 10 kHz. The series resistance (Rs) was <20 MΩ with no compensation.

For mEPSC recordings, glass pipettes were loaded with internal solution containing (in mM) 100 CsMeSO_4_, 25.5 CsCl, 10 HEPES, 8 NaCl, 0.25 EGTA, 10 glucose, 4 MgATP and 0.3 Na_3_GTP (pH 7.3, 280–290 mOsm), and all neurons were held at −70 mV; 50 µM picrotoxin and 0.5 µM TTX were added to block GABA_A_ and Na^+^ currents, respectively.

For the dCA3 local photostimulation experiment, blue light (473 nm, 10 Hz) was delivered through a 200-µm diameter optic fiber which was positioned at the slice surface over the recorded neurons of dorsal CA3.

### Golgi staining

Mice were deeply anesthetized using sodium pentobarbital and then sacrificed. Brains were quickly removed and Golgi staining was performed using the FD Rapid GolgiStainTM Kit (FD NeuroTechnologies, PK401), according to the manufacturer’s instructions. Briefly, after quickly being isolated from each animal whole brains were rinsed once in Milli-Q water and sequentially immersed in impregnation solution (mixed by Solutions A and B) and Solution C. Tissues were then serially cut into sections of 120-mm thickness using a vibratome (Microm, 920120), stained with silver nitrate solution (Solutions D and E), dehydrated through descending alcohol series, and finally mounted with Permount (Thermo Fisher Scientific). Z stack Images of CA3 area were acquired using a microscope (×60 objective lens; BX61; Olympus). We first converted the z stack images to a single image using the Z stack project in ImageJ software. We then chose two to three dendrites from each neuron for density measurement. To measure the density of spines in each dendrite we used the area selection and segmented line tools in ImageJ to obtain the length of each dendrite, and then used Cell Counter in ImageJ to count the numbers of spines in those selected dendrites.

### Behavioral experiments

Adult male mice (aged 2–5 months) were used for all behavior tests. The number of behavioral tests performed in each cohort of mice and the order of behavioral experiments of behavioral tests are described in Supplementary Table 1. Behavioral subjects were individually habituated to the investigator by being handled several times before their first behavior test. On the experimental day, the mice were transferred to the testing room for at least 1 h before behavioral recording. For the stimulation and pharmacogenetic experiments, behavioral testing was performed at least 3–4 weeks following viral injection to allow sufficient time for recovery from surgeries and transgene expression.

### Three-chamber test

The three-chamber apparatus consisted of a Plexiglas rectangular box divided into three equal-sized compartments. In the first session, a test mouse was placed in the middle compartment and allowed to habituate to the apparatus for 10 min. In the second session (sociability test), a stimulus mouse (age- and gender-matched C57BL/6J novel mice) was placed inside a wire containment cup in either the left or right compartment (20 cm × 40 cm × 21 cm). An empty wire containment cup was placed in the other compartment. The test mouse was then left to explore the chambers for 10 min. Time spent by the test mice in investigating each containment cup was measured. The placement of the stimulus mouse on the left or right side of the chamber was systematically alternated between trials to eliminate any chamber bias. In the third session (social preference test), we placed a second stimulus mouse (age- and gender-matched C57BL/6J novel mice) inside an identical wire containment cup in the opposite side chamber, and the test mouse was then left to explore the chambers for 10 min. Time spent by the test mice in investigating the cup containing either the familiar (first stimulus mouse) or the stranger (second stimulus mouse) was calculated. All behavioral videos were recorded using the ANY-maze video tracking system (Stoelting Co.). In the sociability test, the preference index was the time spent exploring the mouse compartment divided by time spent exploring the empty chamber. In the social preference test, the preference index was the time spent exploring the stranger mouse compartment divided by the time spent exploring the familiar mouse compartment.

The stimulation experiments were based on the three-chamber task design described above. The test mice (optical fiber implanted above dCA3) performed the task over 2 days. Half of the test mice were assessed under OFF-laser conditions (the test animal was connected to the fiber-optic patch-cord but no optic stimulation was delivered) on day 1 and ON-laser conditions on day 2. The other half received a counterbalanced protocol. For the social preference test, the test mice did not receive any optic stimulation in the second session (sociability) but did so in the third session where the target zone for light stimulation included all three chambers. As for the encoding, the test mice received optical stimulation in the second session (sociability) but not in the third session. During the ON phases, the optic stimulation was delivered only when the test mice entered the target zone, as defined by the nose-position of the test mice approaching the stimulus mouse.

For chemogenetic experiments, the social behavior tests were performed as the previous test methods with minor modifications. In brief, the mice which had been bilaterally injected in the dorsal CA3 with the virus were housed individually and a stimulus mouse was put into the home cage of the test mice for familiarization for 3 days prior to the test. On the experiment day, the stimulus mice were removed, and the test mice received an i.p. injection of CNO (0.2 mg/kg) or saline. 30 min after the injection, the test mice were placed in the three-chamber social behavior test as described above. For the sociability test, the original stimulus mouse (i.e., familiar mouse) was placed in the left or right compartment inside the wire containment cup, and an empty wire containment cup was placed in the other compartment (compartments systematically alternated). The test mice were allowed to explore the chambers for 10 min. Time spent by each of the test mice in investigating each containment cup was measured. For the social preference test, the stimulus mouse (familiar mouse) was placed in the left or right compartment inside the wire containment cup, and a novel stimulus mouse was placed in the other compartment (compartments systematically alternated). The test mice were allowed to explore the chambers for 10 min. Time spent by each of the test mice in investigating the cup containing either the familiar mouse or the stranger mouse was calculated.

### Resident-intruder test

Male juvenile mice (C57BL/6J, 4 weeks old, male) were used as stimulus mice to reduce the effect of mutual aggression. The test was performed in the home cages of test mice. Testing began immediately when a novel juvenile mouse was introduced to the cage. The test mice were allowed to explore the juvenile mice for 5 min (trial 1), then the juvenile mice were removed. After an inter-trial interval of 1 h, the test mice were re-exposed original (the stimulus mice in trial 1) or a novel juvenile for 5 min (trial 2). Behavioral videos were recorded using the ANY-maze video tracking system (Stoelting Co.). The social behavior (body sniffing, anogenital and nose-to-nose sniffing, following, and allogrooming) initiated by the test subject in the first 1 min in trial 1 and trial 2 was measured. The difference score was calculated by subtracting the time spent in social interaction during trial 2 from the social interaction time during trial 1.

### Open field

The open-field test was used to assess anxiety-related behavior and locomotor activity in an open-field arena (45 cm × 45 cm × 50 cm) under dim light conditions (25 lux). The test mice were individually placed in the center of the chambers and allowed to freely explore for 5 min. The distance traveled in the entire apparatus, as used to evaluate locomotor activity, and the time spent in the central area, as used to estimate the anxiety level, were recorded using an ANY-maze video tracking system (Stoelting Co.).

### Elevated plus maze

The elevated plus maze consisted of a plus-shaped platform with four intersecting arms: two open arms (30 cm × 5 cm, wall-free) and two enclosed arms (30 cm × 5 cm) surrounded by 15-cm-high walls. The maze was elevated 55 cm from the ground. Animals were introduced to the center of the apparatus facing an open arm and then allowed to freely explore the maze for 5 min. Light in the open arms was kept at 40 lux. The time spent in the open arm was recorded using ANY-maze video tracking system (Stoelting Co.).

### Light-dark box test

The light-dark box consisted of one dark compartment (25 cm × 21.5 cm × 26 cm) and one brightly lit compartment (30 cm × 21.5 cm × 26 cm, 350 lux). At the beginning of the experiments, the test mice were individually placed into the dark compartment facing the lit compartment and then were allowed freely explore for 5 min. The time spent in the lit compartment was recorded using the ANY-maze video tracking system (Stoelting Co.).

### Novel object recognition test

This task was performed under the same environmental conditions as the open-field test. This task consisted of a training phase (familiarization phase) and a test phase separated by a 1 h delay. In the training phase, the mice were presented with two objects identical in shape, color, and odor that were placed near the corners of one wall in the arena. Each test mouse was placed in the center of the arena and allowed to explore the objects for 10 min. In the test phase (5 min duration), the animal was placed in the arena and presented with two objects in the same positions as in the training phase: one object being a third copy of the object used in the training phase and the other being a novel object. The positions of the objects in the test and the objects used as either novel or familiar were counterbalanced between the animals within a group and between the control and BWT groups. Behavioral videos were recorded using ANY-maze video tracking system (Stoelting Co.). The time spent exploring the familiar object and novel object was calculated. The novel object preference index was defined as the time spent exploring the novel object divided by time spent exploring the familiar one.

For the stimulation experiments, optical stimulation was administered (optical fiber implanted above dCA3) in the test phase.

### Object-texture-recognition test

This protocol was mostly the same as the “Novel object preference test”, the only difference being that the novel object in the test phase was the same as the familiar object in shape, color, material, and odor but not in texture (i.e., a grooved object).

### The olfactory habituation/dishabituation test

This test was as described previously [36]. In brief, this test consists of sequential presentations of four odors using the cotton swabs: banana, almond, social odor 1, and social odor 2. Each odor was presented three times for a duration of 2 min with an inter-trial interval being 1 min. The olfactory investigation of the cotton swabs was recorded using a stopwatch.

### Forced swim test

Mice were placed for 6 min in a non-escapable cylinder glass tank (30 cm height and 10 cm diameter) filled with water at 22 ± 1 °C to a depth of 10 cm. The immobility time was manually measured only during the last 4 min. The mouse was considered immobile when it remained immobile with only slight movements in order to keep its head above water. After the 6 min test, the mice were removed from the tank, dried off with a towel, and returned to their cage.

### Tail suspension test

In this test, animals were suspended for 6 min at least 50 cm above the floor by adhesive tape placed approximately 1 cm from the tip of the tail, under both acoustic and visual isolation. The immobility time was manually measured only during the last 4 min. The mouse was considered immobile when it hung passively and did not show any movement of the body.

### Real-time place preference

The apparatus consisted of a Plexiglas rectangular box divided into two equal compartments (23 cm × 25 cm × 25 cm). The test mice were allowed to freely move in the box for 20 min. We randomly assigned one side of the chamber as the stimulation side and the counterbalance chamber as the no-stimulation side. The mouse was placed in the non-stimulated side at the onset of the experiment and the optic stimulation was administered to the dCA3 each time the mouse crossed to the stimulation side until it crossed back into the un-stimulation side. The total time spent in either compartment was recorded using the ANY-maze video tracking system (Stoelting Co.).

### Measurement of oxytocin and vasopressin levels

P14 C57BL/6J mice with or without BWT at P12 and P14 Fmr1 KO and littermate control mice were deeply anesthetized with sodium pentobarbital and sacrificed for hippocampal tissue isolation. Then the level of OXT and vasopressin were analyzed respectively through the OXT and AVP ELISA kits (MEIMIAN, China) according to the manufacturer’s instructions.

### qRT-PCR

P14 C57BL/6J mice with or without BWT at P12 and P14 Fmr1 KO and littermate control mice were deeply anesthetized with sodium pentobarbital and then hypothalamic samples were rapidly dissected from the fresh brains. Total RNA was extracted from tissue samples by using TRIzol kit (GENERAY). And total RNA was reverse transcribed into complementary DNA using the PrimeScript^TM^ RT Master Mix (TaKaRa) according to the manufacturer’s protocols. Real-time qPCR was performed using TB Green PreMix Ex Taq (TaKaRa) on CFX-96 (Bio-Rad). The following primers were used: 5′-CTGCCCAGAACATCATCCCT-3′ (forward) and 5′-TGAAGTCGCAGGAGACAACC)-3′ (reverse) for Gapdh; 5′-CCTACAGCGGATCTCAGACTGA-3′ (forward) and 5′-TCAGAGCCAGTAAGCCAAGCA-3′ (reverse) for Oxt. All reactions were repeated in triplicate, and the amount of mRNA was calculated by absolute quantitation.

### Oxytocin treatment

For intraventricular administration, C57BL/6J and Fmr1 knockout mice at P10 were deeply anesthetized using isoflurane and a cannula (RWD life science) was placed into the lateral ventricle (AP, −0.9 mm; ML, 0.4 mm; DV, −2.5 mm). After 2 days, the C57BL/6J mice underwent whisker trimming as described above. For intraventricular injection, OXT was dissolved in ACSF to achieve a final concentration of 10 μg/ml. Then, 2 μl of OXT or ACSF (the amount of OXT was 0.02 μg per mouse, 2 μg/kg) was injected into the lateral ventricle by using a micro syringe pump at a rate of 500 nl/min in C57BL/6J (with whisker trimming) and Fmr1 knockout mice (P12). The injections were given twice per day at intervals of 12 h from P12 to P16. After 2 months, the mice underwent the classic three-chamber social behavior test and mEPSC recording. For intranasal administration, in C57BL/6J pups (injected with lentivirus at P7) and Fmr1 KO pups, OXT was dissolved in saline (0.9% NaCl) to achieve a final concentration of 400 μg/ml and then a drop of 2.5 μl OXT solution was gently placed equally on both nostrils of each mouse (100 μg/kg). The administration was given twice per day at an interval of 12 h from P12 to P16 and the social behavior test was then performed after 2 months. In adult Fmr1 KO and BWT mice (early life BWT), OXT was dissolved in saline (0.9% NaCl) to achieve a final concentration of 500 μg/ml, and each mouse received 2 × 2.5-μl drop (100 μg/kg). For the chronic intranasal treatments, the administration was given twice per day at an interval of 12 h for constitute 5 days. The social behavior test was then performed 24 h after the last administration. For the acute intranasal treatments, mice were administered with OXT solution just once (100 μg/kg), 30 min before the test. Each mouse received 2 × 2.5-μl drops.

### Western blot

Dorsal CA3 tissues (1 mg) from P12 control and shRNA-virus treated mice were homogenized in RIPA lysis buffer (Yeasen, Shanghai, China) containing cOmplete™ EDTA-free Protease Inhibitor Cocktail (Roche Applied Science, Basel, Switzerland) and disrupted by ultra-sonication. Protein samples were separated on 10% tris-glycine gels before being transferred onto PVDF (0.45 μm, Merk Millipore, Darmstadt, Germany) and the PVDF was then blocked in 5% nonfat milk. Immunoblotting was conducted with HRP-conjugated secondary antibodies and ECL Western Blotting Substrate (BioSharp, Beijing). The following primary antibodies were used in this study: rabbit anti-Oxtr (Abclonal, Wuhan, China) and rabbit anti-β-tubulin (Yeasen, Shanghai, China). Loading controls were run on the same gel. Signals were acquired using Amersham Imager 600 (GE Life Science, Boston, USA), and images were analyzed using Fiji ImageJ software.

### Single-unit recording and data analysis

Modified drivable electrode arrays were implanted in the dCA3. The electrode arrays consisted of seven nichrome tetrodes of four thin entwined wires (California Fine Wire Co., Grover Beach, CA, USA). The micro-wire bundle was attached to a home-made micro-drive. One ground wire was soldered to a 32-channel connector (Omnetics Connector Corp., Minneapolis, MN, USA). Mice were allowed to recover for at least 5 days and then the electrode arrays were connected to a 32-channel preamplifier head-stage (Plexon Inc., Dallas, TX, USA). Mice were recorded for two sessions per day (details are described in the three-chamber test methods) and electrodes were advanced by ~60 μm at the end of the recording. During sessions, the behavior and all signals recorded from each tetrode were amplified and filtered between 0.1 Hz and 10 kHz, and spike waveforms were digitized at 40 kHz. Spikes were sorted using the software Offline Sorter (Plexon). Units were accepted only if a distinct cluster was visible in a two-dimensional plot of the two largest principal components. In total, 5 control mice and 7 BWT mice at the age of 2–3 months were implanted with electrodes and used for data collection. Neurons with mean firing rates >0.5 Hz were included in the analysis. Then analysis was performed using MATLAB, referencing neural activity to behavior. A given neuron that significantly responded to a defined behavior would be reflected by its reliable responses across different trials. The responses of each neuron were averages of 2–20 behavior trials. We then calculated the averaged response during the social investigation, based on the *z*-scored PSTHs. For each recorded unit, PSTHs were *z*-score transformed by subtracting the mean firing rate and dividing by the standard error of each unit’s firing rate. To test the significance of changes in firing rate, we used individual unit analysis. A non-parametric Wilcoxon signed-rank test was conducted on each unit to determine whether the mean firing rate during the event was significantly different from baseline (from –2 s to –1 s), and units were classified into three populations: excited, inhibited, or unchanged. The responses and relative proportions of behavior-excited, behavior-inhibited, and behavior-no-response units before and during the events were compared using a *t*-test.

### Immunohistochemistry and imaging

Mice were deeply anesthetized with sodium pentobarbital and then sacrificed. Intracardial perfusion was then conducted with 0.9% saline followed by 4% paraformaldehyde in 0.1 M phosphate buffer (PBS). The brain was removed and placed in 4% paraformaldehyde at 4 °C for 6–8 h and then transferred to 30% sucrose solution for at least 36 h at 4 °C. Coronal brain sections were cut using a freezing microtome (Leica) at 30 or 40 µm. For c-Fos staining, sections were washed three times in 0.01 M PBS and rinsed with 0.3% Triton X 100 in 0.1 M PBS (30 min) or frozen methanol (10 min at −20 °C), then blocked with 10% normal bovine serum for 1 h at room temperature. Sections were incubated with the primary antibodies: anti-c-Fos (1:800, gp, Sysy) overnight at 4 °C. After rinsing, sections were incubated with fluorophore-conjugated secondary antibody for 2 h at room temperature (1:1000; Millipore). The nuclei were stained with DAPI and the slices were mounted on microscope slides. Images were captured using an Olympus FV-1200 (×10) inverted confocal microscope. For OXT staining, sections were pretreated in 0.3% hydrogen peroxide for 10 min to block endogenous peroxidase activity and blocked in PBS containing 1% goat serum albumin and 0.3% Triton X 100 for 1 h at 37 °C. Sections were then incubated with OXT antibody (1:2000, Phoenix Pharmaceutics, H-051–01) overnight at 4 °C. Sections were subsequently washed and then incubated with biotinylated secondary antibodies (undiluted, SP-9001, ZSGB BIO) for 2 h at 37 °C, followed by application of horseradish enzyme-labeled streptavidin (undiluted, SP-9001, ZSGB BIO) for 2 h at 37 °C. Immunoreactivity was visualized using a DAB Substrate Kit (D0426, Sigma Aldrich). Images were acquired with an Olympus VS120 (×10) virtual slide scanning system.

For quantification of *c-Fos*- and OXT-positive cells, 12 sections from 4 mice (3 sections per mouse) were used. Cell counts and analysis were performed using the ImageJ software.

### Statistics

Sample sizes were chosen based on previous studies conducted in the same field. In all experiments, data acquisition and analyses were performed blindly. All results are presented as the mean ± SEM. GraphPad Prism was used for all statistical analyses. All data sets were first tested for normality before choosing the statistical test. Statistical significance was assessed by unpaired Student’s *t*-test, paired Student’s *t*-test, Mann–Whitney *U* test, Wilcoxon matched-pairs signed-rank test, and two-way ANOVA where appropriate. The significance level was set at *P* < 0.05 (*P < 0.05, **P < 0.01, ***P < 0.001).

## Results

### Early life whisker trimming selectively affects social discrimination in adulthood

**Fig. 1:**
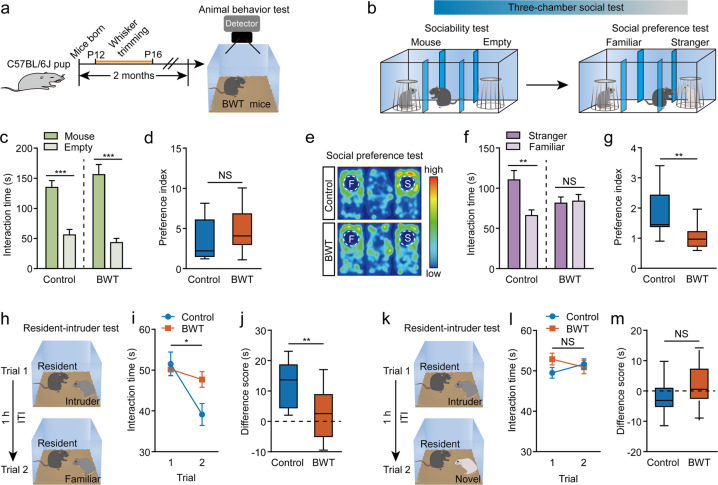
Bilateral whisker trimming through P12–16 dramatically impairs social discrimination in adulthood. **a** Experimental strategy diagram. **b** Diagram showing the three-chamber test. **c**, **d** Sociability test. Time spent (**c**, Control: *n* = 11 mice, *P* = 0.0002; BWT: *n* = 14 mice, *P* < 0.0001; paired *t*-test) and preference (**d**, *P* = 0.2636, unpaired *t*-test) for sniffing the mouse chamber or the empty chamber. **e**–**g** Social discrimination test. **e** Representative heatmaps. Letter F indicates the familiar mouse and letter S indicates the stranger mouse. **f**, **g** Social discrimination in the three-chamber test showing time spent (**f**, Control: *P* = 0.0011; BWT: *P* = 0.7351; paired *t*-test) and preference (**g**, *P* = 0.0014, Mann–Whitney *U* test) for interacting with a stranger mouse versus a familiar mouse. **h**–**m** Resident-intruder test. The same (**h**) or different (**k**) intruder mouse was used in the two trials. **i**, **j** Unlike control mice, BWT mice failed to display decreased investigation time during trial 2 of an identical intruder (**i**, *P* = 0.0289, two-way ANOVA; **j**, *P* = 0.0090, unpaired *t*-test. Control: *n* = 9 mice; BWT, *n* = 13 mice). **l**, **m** The control and BWT group explored two different mice similarly (**l**, *P* = 0.1590, two-way ANOVA; **m**, *P* = 0.1309, unpaired *t*-test. Control: *n* = 11 mice; BWT: *n* = 13 mice). ITI inter-trial interval. *P < 0.05; **P < 0.01; NS, not significant. Data presented as mean ± SEM.

To explore the role of early life sensory experience on adult brain function and/or behavior, we performed BWT during a short period in early life from postnatal 12 (P12) to P16 in male C57BL/6J mice. We then let the whiskers re-grow naturally from that point onwards. We tested any behavioral alterations in those mice when they reached 2 months of age (Fig. 1a). Interestingly, we observed deficits of social behavior in the adult BWT mice. In a three-chamber sociability test [37] examining the preference of a subject mouse for a chamber containing a mouse versus an empty chamber, both the control mice (without whisker trimming experience) and the BWT mice displayed similar and significant increased interest in the chamber containing a mouse (Fig. 1b–d). However, the BWT group exhibited obvious deficits in the three-chamber social preference test compared to the controls. Unlike the control mice that spent significantly increased time interacting with the novel, unrelated, mouse compared with the time they spent interacting with a familiar mouse, the BWT mice exhibited no such interaction preference between a novel mouse and a familiar one (Fig. 1e, f). Furthermore, the preference index (time spent exploring the stranger mouse compartment divided by time spent exploring the familiar mouse compartment) was significantly less for the BWT group than that in the control group (Fig. 1g). These results indicate that BWT during P12–16 had impaired social discrimination, but not affected sociability in adulthood. We further verified our observations using a resident-intruder test [31]. In trial 1, a subject mouse was exposed to an unfamiliar mouse for an interaction time of 5 min. Then, after a 1-h inter-trial interval, either the previous mouse as encountered in trail 1, which should be now considered as familiar, or a second novel mouse was introduced to the subject mouse in trial 2 (Fig. 1h–m). A significantly reduced time was spent exploring the familiar mouse for control mice, but not for BWT mice. However, the control mice and BWT mice showed no differences in time spent exploring the novel mouse. These results suggested that unlike control mice, BWT mice failed to recognize the familiar mouse that had interacted with them 1 h previously, and indicating an impairment in the social memory of these mice (Fig. 1i–m).

It is important to consider if the deficit in the social function of BWT mice may be due to other reasons, such as impaired ability to recognize novel odorants (since odorant information is critical for social behavior in mice [38]) or the loss of interest in novel objects, or reduced tactile perception sensitivity. To exclude these possibilities, we firstly performed odorant cue recognition experiments and found that the BWT mice displayed reduced interest when repeatedly exposed to the same odorant, which included responses to both non-social and social odorants, but significantly increased the exploring time when exposed to a new odorant (Supplementary Fig. S1). In this test the BWT mice behaved in a manner similar to control mice, suggesting that BWT mice have retained normal olfactory discrimination ability. We next found that the BWT mice had normal preferences for novel objects, as showed in the novel-object-recognition assay (Supplementary Fig. S2a–c), indicating the impairment of social discrimination was not due to a lack of interest in novelty. Another noted similarity was in the object-texture-recognition assay where both the control and BWT mice groups spent more time exploring the novel object when the only difference was surface roughness (Supplementary Fig. S2d–f). This suggests that precise tactile perception had remained un-altered in these BWT mice. These results strengthen the understanding that the deficiency in BWT mice is specific to social novelty, but does not include any discrimination deficiencies relating to novel objects. We next wanted to test whether the social discrimination deficits in the BWT group were attributable to the early life sensory experience of manipulation causing stress, such as anxiety or depression, in later adulthood. In anxiety-related behavioral assays including the open-field test (Supplementary Fig. S3a–c), elevated plus maze (Supplementary Fig. S3d, e), and light/dark preference (Supplementary Fig. S3f, g) tests, and in depression-related behavioral assays such as forced swimming (Supplementary Fig. S3h) and tail suspension (Supplementary Fig. S3i), no differences were observed between BWT and control groups. This indicated that BWT mice were neither more anxious nor more depressed than the control mice in adulthood.

To test whether the timing of whisker trimming was important, we performed BWT in different time windows and then tested the behavioral outputs of mice in adults. We first performed BWT for 5 consecutive days in 2-month-old C57BL/6J mice, then tested their social behavior (Supplementary Fig. S4a–c). We found that 5-day whisker trimming during adulthood failed to produce any effect for either the sociability test or the social preference test (Supplementary Fig. S4b, c). Next, we performed BWT of B6 mice from P9–16 and from P16–20, then let the whiskers re-grow naturally from that point onwards and test social behaviors when those mice had reached 2 months of age. We found that whisker trimming during P16–20 failed to induce any social behavior dysfunction (Supplementary Fig. S4d, e). However, in line with the results of whisker trimming through P12–16, social discrimination performance, but not sociability performance, was significantly impaired in mice that underwent whisker trimming during P9–16 (Supplementary Fig. S4d, e). These results indicate the precise timing if whisker trimming is important for such behavioral outputs.

### Hippocampal dorsal CA3 was aberrantly activated in adult BWT mice during the social discrimination test

**Fig. 2:**
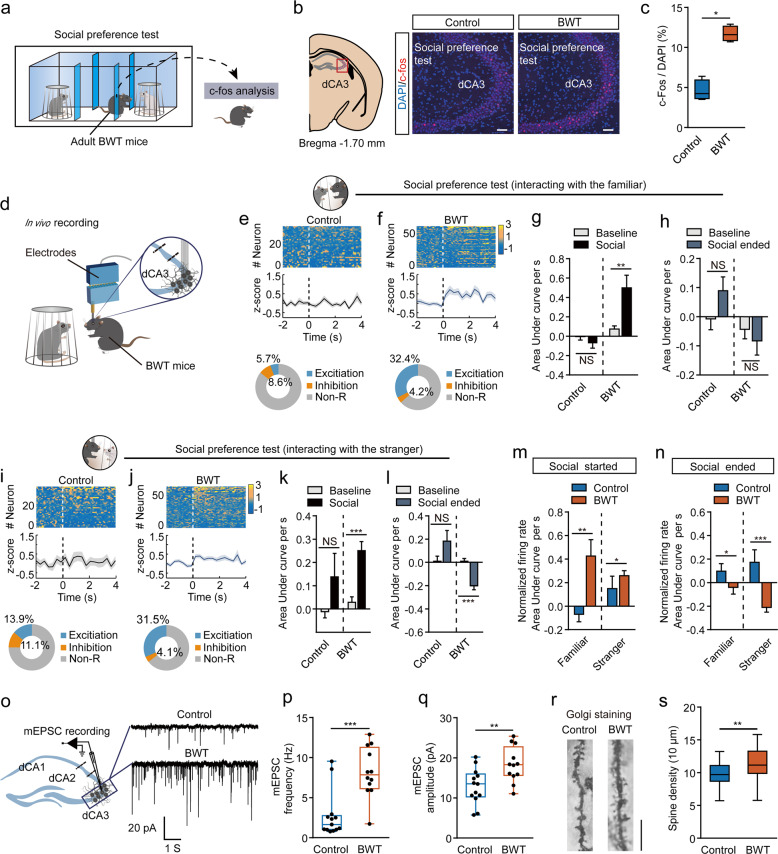
Social discrimination deficit in BWT mice is associated with aberrant activation of hippocampal dCA3. **a** Schematic of c-Fos analysis after social discrimination test. **b** Representative images of c-Fos expression. Scale bars, 50 μm. **c** Quantification of the numbers of cells expressing c-Fos. *N* = 4 mice, each group; *P* = 0.0286, Mann–Whitney test. **d** Diagram showing the tetrode recording strategy. Control: *n* = 35 neurons from 5 mice; BWT: *n* = 71 neurons from 8 mice. **e**–**h** Tetrode recording of interaction with the familiar mouse. **e**, **f** Heatmaps of normalized *z*-scored activity (**e** and **f** upper), average activity (**e** and **f** middle) and relative proportions of neurons (**e** and **f** lower). **g**, **h** Mean population activity (area under the *z*-score curve) before, during (**g**, Control: *P* = 0.4413; BWT: *P* = 0.0008), and after (**h**, Control: *P* = 0.1227; BWT: *P* = 0.0943) social interaction. Each group, Wilcoxon matched-pairs signed-rank test. **i**–**l** Tetrode recording of interaction with the stranger mouse. **i**, **j** Heatmaps of normalized *z*-scored activity (**i** and **j** upper), average activity (**i** and **j** middle) and relative proportions of neurons (**i** and **j** lower). **k**, **l** Mean population activity (area under the *z*-score curve) before, during (**k**, Control: *P* = 0.5014; BWT: *P* = < 0.0001), and after (**l**, Control: *P* = 0.1898; BWT: *P* < 0.0001) social interaction. Each group, Wilcoxon matched-pairs signed-rank test. **m**, **n** The normalized firing rate of recorded neurons related to where social interaction began (**m**, familiar: *P* = 0.0034; Stranger: *P* = 0.0209) and ended (**n**, familiar: *P* = 0.0261; Stranger: *P* = 0.0004). Each group, Mann–Whitney *U* test. **o** Representative mEPSC recordings from control mice (upper) and BWT mice (lower). **p**, **q** mEPSC frequencies (**p**, *P* = 0.0009, Mann–Whitney *U* test) and mEPSC amplitudes (**q**, *P* = 0.0053, unpaired *t*-test). Control, *n* = 13 neurons from 4 mice; BWT, *n* = 12 neurons from 4 mice. **r** Sample images showing the morphology of dendritic spines. Scale bar, 10 μm. **s** The spine density of BWT mice was significantly increased. Control: *n* = 34 neurons from 6 mice; BWT: *n* = 31 neurons from 6 mice; *P* = 0.0079, unpaired *t*-test. *P < 0.05; **P < 0.01; ***P < 0.001; NS, not significant. Error bars indicate SEM.

We next sought to explore the cause of social discrimination deficit in BWT mice. And 1.5 h after the social preference test, C57BL/6J mice, with or without early life bilateral whisker trimmed experience, were sacrificed and brain slices were cut for early gene *c-Fos* immunostaining (Fig. 2a). We observed comparable *c-Fos*-positive cells in different hippocampal areas including the dCA1, dCA2, dDG, and vCA1 (Supplementary Fig. S5) between BWT and control mice. However, BWT mice displayed significantly more *c-Fos*-positive cells compared to the control groups in the dCA3 area (Fig. 2b, c). This result indicated that in BWT mice which had also displayed social discrimination deficit, the dCA3 area had been aberrantly activated during the social preference test.

To further verify that the over-activation of dCA3 was correlated to abnormal social interaction in BWT mice in vivo, we performed single-unit recordings within the dCA3 of freely moving mice. Mice with chronically implanted recording electrodes into the dorsal CA3 pyramidal layer were used for three-chamber social interaction tests (Fig. 2d). Principal component analysis of the single-unit activity in relation to social interaction in the initial sociability test revealed comparable cell firing patterns between the BWT group and control group (Supplementary Fig. S6). These results were consistent with the fact that the sociability of BWT mice had remained unchanged. However, in the social preference test, we observed a significantly higher increase in one population of neurons that were activated during social interaction bouts with a familiar mouse (32.4% in BWT and 5.7% in control) or a stranger mouse (31.5% in BWT and 13.9% in control) from the BWT group as compared to the control group (Fig. 2e–n). The area under the curve analysis also revealed that during social interaction bouts with both familiar and stranger mice the firing rate of dCA3 cells had increased dramatically in the BWT group but not in the control group (Fig. 2g, k, m). It is also important to note that the CA3 neuronal activity had been rapidly enhanced during the period where the object mice were attempting to interact with any mouse, no matter whether the mouse was familiar or a stranger. This immediately decreased to normal firing levels when the social interaction ended (Fig. 2h, l, n and Supplementary Movies S1–S4). Therefore, consistent with the activity-dependent early gene mapping results, our in vivo recording data revealed that dCA3 cells of BWT mice had been temporarily over-activated during social preference tests during both explorations of either a familiar mouse or a stranger mouse.

We then wanted to explore the mechanisms leading to the over-activation of dCA3 cells. Using whole-cell patch recording of acute brain slices we observed a significant augmentation of spontaneous mEPSC frequencies and amplitudes in the BWT group (Fig. 2o–q). We then performed Golgi staining to test the morphological features of the dendrites of dCA3 cells. In line with the electrophysiological recording results, we found there to be more dendritic spines in BWT mice as compared to control mice (Fig. 2r, s). These results indicated that the dCA3 cells in BWT mice had received more synaptic inputs than those of the control mice.

Next, we wanted to investigate whether early life whisker experience deprivation alters CA3 activity at younger ages. We performed whisker trimming during P12–16, and recorded dCA3 mEPSC at P17. We observed a significant increase in the mEPSC frequency, but not amplitude when compared to P17–18 mice that had not experienced whisker trimming (Supplementary Fig. S7), suggesting that BWT during P12–16 had begun to affect dCA3 activity at an early age.

### Manipulation of dCA3 neuronal activity could bi-directionally modulate social behavior

**Fig. 3:**
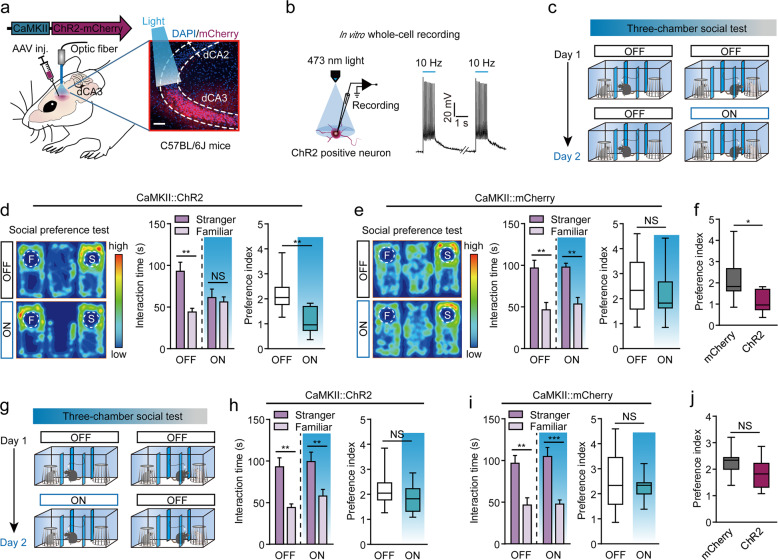
Optogenetic activation of dCA3 in naive mice leads to deficit in social discrimination. **a** Diagram of viral injection and fiber implantation strategy for photoactivation of dCA3 neurons in C57BL/6J mice. Scale bar, 80 μm. **b** ChR2-expressed neurons were activated by blue light stimulation (473 nm,10 HZ). **c** The behavioral design of the social discrimination test in a three-chamber task during photostimulation of dCA3 neruons in C57BL/6J mice. **d** ChR2-expressed mice in social discrimination test. Representative heatmaps for the ChR2-expressed group (**d** left). When receiving blue light stimulation, the ChR2-expressed group showed significantly decreased time spent interacting with the novel mouse (**d** middle, OFF: *P* = 0.0039, Wilcoxon matched-pairs signed-rank test; ON: *P* = 0.5968, paired *t*-test) and preference index (**d** right, *P* = 0.0021, unpaired *t*-test) for a stranger mouse, when compared to those before light stimulation. *N* = 9 mice. **e** Social preference test from mCherry-expressed group. Representative heatmaps for mCherry-expressed group (**e** left). The interaction time (**e** middle, OFF: *P* = 0.0033; ON, *P* = 0.0011; paired *t*-test) and preference index (**e** right, *P* = 0.5407, unpaired *t*-test) for the mCherry-expressed group did not show significant changes from before to after blue light stimulation. *N* = 9 mice. **f** The preference index for a stranger mouse. *P* = 0.0131, unpaired *t*-test. **g** Blue light was delivered to dCA3 only during the familiarization period. **h** The ChR2-expressed mice spent more time interacting with the stranger mouse than the familiar mouse regardless of the presence or absence of photostimulation in the prior familiarization period (**h** left, OFF: *P* = 0.0039, Wilcoxon matched-pairs signed-rank test; ON: *P* = 0.0017, paired *t*-test) and the preference index did not reveal any significant changes (**h** right, *P* = 0.2721, unpaired *t*-test). *N* = 9 mice. **i** The mCherry-expressed group spent more time exploring the stranger mouse than the familiar mouse (**i** left, OFF: *P* = 0.0033; ON: *P* < 0.0001; paired *t*-test). The preference index did not show significant changes for the mCherry-expressed group (**i** right, *P* = 0.9090, Mann–Whitney *U* test). *N* = 9 mice. **j** The preference index for interacting with a stranger mouse. *P* = 0.1140, unpaired *t*-test. *P < 0.05; **P < 0.01; NS, not significant. Data presented as mean ± SEM.

To elucidate any direct linkage between the augment function of dCA3 and social discrimination deficit we next manipulated the neural activity of dCA3 through both optogenetic and chemogenetic methods. We first targeted pyramid cells through a viral construct with CaMKIIα as promoter coding for channelrhodopsin fused to mCherry fluorescent protein (AAV-CaMKIIα-hChR2-mCherry) [39, 40], into the dCA3 region of naive C57BL/6J mice and positioned optical fibers above the dCA3 region for the selective activation those local neurons (Fig. 3a). Light stimulation evoked action potentials through whole-cell patch recording in the acute brain slices confirming the expression of ChR2 in the dCA3 principal neurons (Fig. 3b). Next, we initiated three-chamber social interaction tests, with or without light stimulation, over 2 consecutive days for ChR2-expressed and mCherry-expressed control mice (Fig. 3c). On day 1, without photostimulation, we executed sociability (also referred to as the familiarization period) and then conducted social preference tests. We found that mice with the expression of ChR2 spent remarkably more time exploring and interacting with a novel mouse than with a familiar one, indicating normal social discrimination. However, on day 2, we conducted a social preference test accompanied by light stimulation throughout the testing episode. In this case, the ChR2-expressed mice lost their preference for interacting with a strange/ unfamiliar mouse (Fig. 3d). This phenomenon was not reproduced in mCherry-expressed mice, even though we also applied light stimulation during the whole period of the social preference test (Fig. 3e, f).

Next, we wanted to discover if the over-activation of dCA3 cells only occurring during the familiarization period would be capable to disrupt later social novelty recognition. To test this, we did not apply any light stimulation on day 1 and only applied light stimulation during the familiarization period but not during the social preference test period on day 2 (Fig. 3g). We found that on day 2 the ChR2-expressed group displayed a significant preference toward interaction with an unfamiliar mouse over a familiar mouse during social preference test, a similar result to the mCherry-expressed group. This indicated that light stimulation in the familiarization period had failed to impair social discrimination (Fig. 3h–j). However, we needed to consider if the optogenetic activation of dCA3 may cause some secondary effects on mice, such as reducing their interest toward novel objects or inducing place preference, therefore affecting social discrimination indirectly. We were able to exclude these possibilities by finding that the light stimulation of dCA3 did not provoke any changes in their performance in the novel-object-recognition test (Supplementary Fig. S8a–c), nor induce any changes in preference or avoidance toward the chamber with light stimulation in the real-time place preference test (Supplementary Fig. S8d–f). Taken together, these results suggest that the over-activation of dCA3 during the social preference test period was sufficient to impair social discrimination in naive mice.

**Fig. 4:**
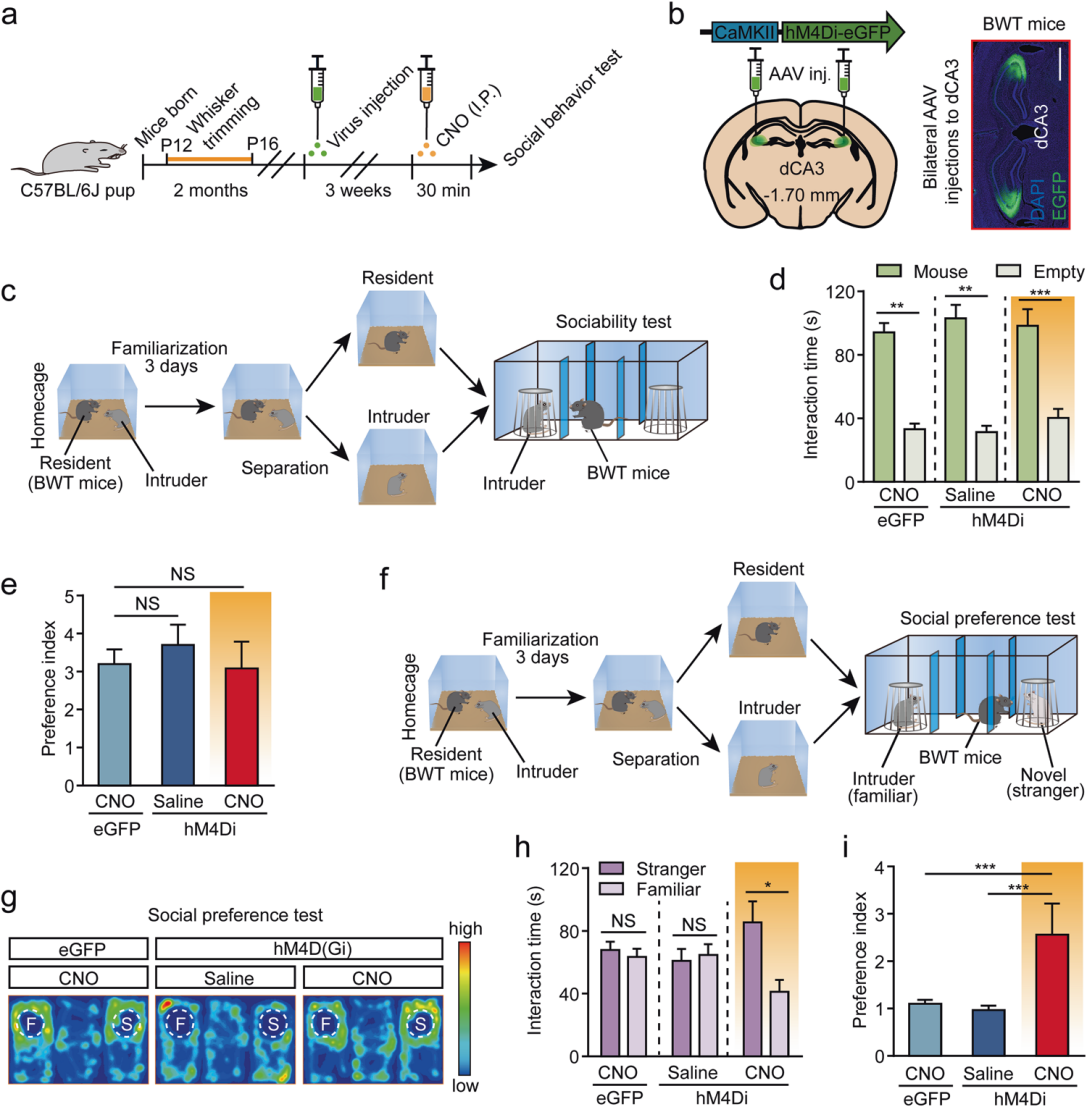
Chemogenetic silencing of dCA3 rescues social discrimination deficits in mice that had undergone bilateral whisker trimming during P12–16. **a** Behavioral schedule. Two-month-old mice with bilateral whisker trimming experience during P12–P16 were bilaterally injected with AAV-CaMKIIα-hM4Di-EGFP (or eGFP alone) into the dCA3. The social behavior test was then performed. **b** dCA3 confocal image from a BWT mouse indicating the placement of the virus expression. Scale bar, 1 mm. **c**–**e** Sociability test. **c** Protocol for sociability test. **d**, **e** Sociability in the three-chamber test showing time spent (**d**) and preference (**e**) for sniffing the mouse chamber or the empty chamber. CNO (0.2 mg/kg) was delivered half an hour before behavioral tests. **d** CNO (eGFP): *n* = 11 mice, *P* = 0.0010, Wilcoxon matched-pairs signed-rank test; Saline (hM4Di), *n* = 9 mice, *P* = 0.0039, Wilcoxon matched-pairs signed-rank test; CNO (hM4Di), *n* = 9 mice, *P* = 0.0005, paired *t*-test. **e** CNO (eGFP) vs CNO (hM4Di), *P* = 0.4023, Mann–Whitney *U* test; Saline(hM4Di) vs CNO(hM4Di), *P* = 0.3347, Mann–Whitney *U* test. **f**–**i** Social preference test. **f** Protocol for social preference test. **g** Representative heatmaps from the social preference test. **h**, **i** Time spent interacting with the stranger mouse (**h**) and the preference index for the stranger mouse (**i**) showed significant increases via chemogenetic silencing of dorsal CA3 neurons for BWT mice. **h** CNO (eGFP): *n* = 11 mice, *P* = 0.3997, paired *t*-test; Saline (hM4Di): *n* = 9 mice, *P* = 0.5095, paired *t*-test; CNO (hM4Di): *n* = 9 mice, *P* = 0.0103, paired *t*-test. **i** CNO (eGFP) vs CNO (hM4Di), *P* = 0.0008, Mann–Whitney *U* test; Saline (hM4Di) vs CNO (hM4Di), *P* = 0.0008, Mann–Whitney *U* test. *P < 0.05; ***P < 0.001; NS, not significant. Data presented as mean ± SEM.

Next, we wanted to test whether the inhibition of the activity of dCA3 was capable to rescue social discrimination deficit in BWT mice. To answer this we expressed hM4Di by injection of the AAV-CaMKIIα-hM4Di-eGFP virus into bilateral dCA3 of BWT mice and then allowed the mice to recover for 3 weeks. We then initiated the three-chamber social test 30 min after the mice had received CNO or saline treatments (Fig. 4a–c). Compared to saline treatment, hM4Di-expressed BWT mice receiving CNO treatment showed no altered preferences when presented with a mouse vs an empty chamber (Fig. 4d, e), but recovered their interest and spent significantly more time exploring an unfamiliar mouse than exploring a familiar mouse during the social preference test (Fig. 4f–i). However, CNO treatment alone, for example in eGFP-expressed BWT mice, failed to rescue the social discrimination deficit (Fig. 4g–i).

We then wanted to discover what downstream nucleus of dCA3 in the social discrimination circuits is. Since the CA3 to CA1 projection has been extensively studied and a recent study has suggested that memory engram cells of ventral CA1 (vCA1) are crucial for social memory [30], we tested the possible role of the dCA3-vCA1 pathway. We targeted the AAV-CaMKIIα-ChR2-eGFP virus into dCA3 area of naive C57BL/6J mice and positioned optical fibers above the vCA1 for manipulation of the dCA3-vCA1 pathway during the social preference test (Supplementary Fig. S9a, b). We found that the photo excitation of the dCA3-vCA1 pathway was sufficient to reduce the interaction time remarkably of the test mouse to a stranger mouse in the social preference test, and resulted in decreases in the preference index (Supplementary Fig. S9c–e). Meanwhile, such manipulation failed to alter novel object recognition (Supplementary Fig. S9f–h). These results suggested that vCA1 is the downstream nucleus of dCA3 in social behavior circuitry.

### Reduced oxytocin levels via early life whisker trimming contributed to aberrant over-activation of dCA3 and social discrimination deficit in BWT mice

**Fig. 5:**
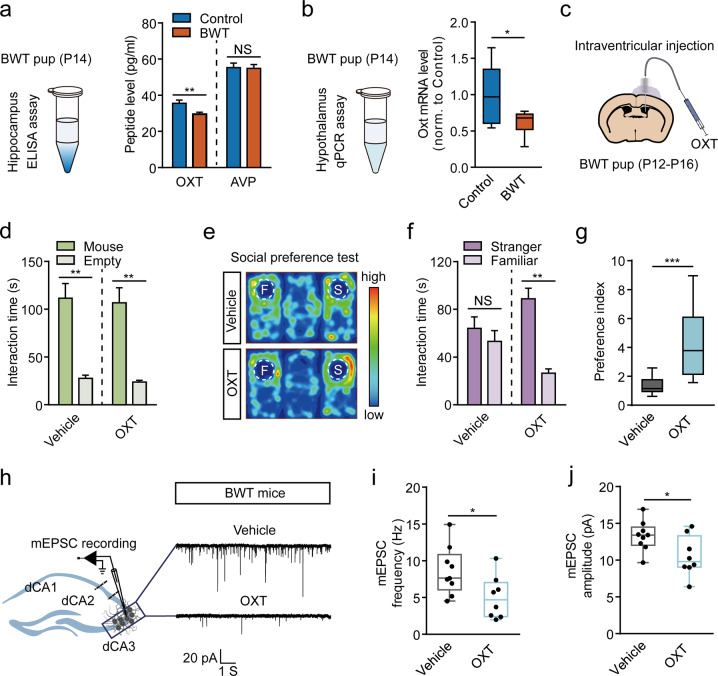
Early life oxytocin treatment rescues social discrimination deficit in adult BWT mice. **a** Left, diagram shows the peptide ELISA assay. Right, oxytocin (OXT) and vasopressin (AVP) peptide levels in hippocampus (control: *n* = 19 mice; BWT: *n* = 20 mice. OXT: *P* = 0.0174, Mann–Whitney *U* test. AVP, *P* = 0.8851, unpaired *t*-test). **b** Oxytocin mRNA level in the hypothalamus in P14 mice with or without whisker trimming at P12 (control: *n* = 6, BWT: *n* = 7; *P* = 0.0449; unpaired *t*-test). **c** Schematic shows intraventricular injection of OXT. **d** Time spent sniffing the mouse chamber or the empty chamber in sociability test (vehicle: *n* = 9 mice, *P* = 0.0039;. OXT: *n* = 11 mice, *P* = 0.0010; Wilcoxon matched-pairs signed-rank test). **e**–**g** Social preference test. **e** Representative heatmaps. **f**, **g** Time spent (**f**, Vehicle: *n* = 9 mice, *P* = 0.2369, paired *t*-test. OXT: *n* = 11 mice, *P* = 0.0010, Wilcoxon matched-pairs signed-rank test) and preference (**g**, *P* = 0.0008, Mann–Whitney *U* test) for interacting with a stranger mouse versus a familiar mouse. **h** Representative spontaneous mEPSC recordings in dCA3 cells from vehicle (upper) and OXT groups (lower). **i**, **j** mEPSC frequencies (**i**, *P* = 0.0306) and mEPSC amplitudes (**j**, *P* = 0.0304) in dCA3 (vehicle, *n* = 9 neurons, *N* = 3 mice; OXT, *n* = 8 neurons, *N* = 3 mice). Unpaired *t*-test. *P < 0.05; **P < 0.01; ***P < 0.001; NS, not significant. Data presented as mean ± SEM.

After revealing a direct link between aberrant activation of dCA3 and impaired social discrimination in BWT mice, we next wanted to investigate how whisker trimming during early development enhanced synapses formed by dCA3 neurons. A recent study suggested that early life vision or whisker deprivation reduces the brain OXT level and modifies synapse development in cortical areas [19]. In addition, given that a large number of studies have suggested the crucial role of OXT in social interaction [26, 41, 42], we hypothesized that OXT might be involved in the social discrimination deficit caused by over-activation of dCA3 in BWT mice. To test this hypothesis, we first collected hippocampal samples in P14 mice with or without whisker trimming experience at P12. Then, using an ELISA assay [43], we found that the 2-day previous whisker trimming had significantly reduced the hippocampal OXT, but not the vasopressin levels (Fig. 5a). Furthermore, we performed qRT-PCR of the hypothalamus from BWT and control P14 mice to detect the mRNA levels of OXT. We consistently observed a significant reduction of OXT mRNA level in BWT mice (Fig. 5b). We also counted the number of OXT-expressing cells in the PVN and SON of P14 BWT and control mice and did not find any changes (Supplementary Fig. S10). Next, we wanted to explore whether an early life intracerebroventricular injection of OXT could rescue the social behavior deficit in BWT mice. We first implanted a cannula into the right lateral ventricle at P10 and performed whisker trimming at P12 as described previously. After daily intracerebral injections of OXT, with ACSF as control, during P12 to P16, we then waited until mice were 2 months old (Fig. 5c). Interestingly, a brief period of OXT treatment resulted in the recovery of the social discrimination deficit in adult BWT mice. While no differences were observed in the sociability test between mice that received OXT and vehicle treatments (Fig. 5d), mice receiving OXT treatment spent significantly more time interacting with a stranger mouse in the three-chamber test (Fig. 5e–g). Furthermore, the rescued social discrimination deficit was seen to correlate with a reduced dCA3 spontaneous mEPSC frequency and amplitude in adult BWT mice that had undergone early life OXT treatment (Fig. 5h–j). These results suggest that early life OXT treatment is sufficient to remove the redundant synapses formed by dCA3 neurons and effectively prevent the social discrimination deficit in BWT mice.

To further investigate whether OXT treatment directly plays a role in reducing the neural activity of dCA3 and recovering the social deficits, we knock down the OXT receptors in the dCA3 of neonatal C57BL/6J mice. We first constructed a lentivirus expressing Oxtr siRNA or control siRNA, then bilaterally injected it into the dorsal CA3 of mice at P7. The knockdown efficiency of Oxtr siRNA construct was tested at P12 by western blot analysis using the tissue samples collected from hippocampal dorsal CA3 (Supplementary Fig. S11a, b). Neonatal knockdown of OXT receptor in dCA3 mimicked the effects of early life BWT, resulting in impaired social discrimination preference and enhanced dCA3 neural activity. In addition, the social dysfunction caused by neonatal Oxtrs knockdown could not be rescued by concurrently early life OXT treatment (Supplementary Fig. S11c–i). These results suggested that manipulation of social behavior by neonatal OXT treatment was acting on the Oxtr mediated signaling pathway in the dCA3.

Next, we wanted to ask whether adult OXT treatment in BWT mice could rescue their social dysfunction through suppressing dCA3 neural activity. Since a number of studies have suggested intranasal administration of OXT as an efficient way to deliver it into the brain [26], we performed intranasal administration instead of intracerebroventricular injection of OXT. We found that BWT mice recover their social behavior dysfunction 30 min after a single dose of intranasal OXT treatment, as indicated by BWT mice preferring to explore a novel object in the social preference test phase of the three-chamber test (Supplementary Fig. S12a–d). To test whether OXT treatment in adulthood has a long-lasting effect on social behavior performance, we performed 5 constitutive days of intranasal OXT daily treatment on BTW mice, and then tested the social behaviors 24 h after final OXT treatment. However, we observed that the rescue effects of OXT on social behavior dysfunction in BWT mice had disappeared 24 h after OXT treatment (Supplementary Fig. S12a–d). These results indicated that the rescue effects of social dysfunction are temporal, and fail to last beyond 24 h. We also performed whole-cell patch recording of dCA3 mEPSC in adult BWT mice 30 min after receiving OXT treatment and found no differences when compared to mice receiving saline treatment (Supplementary Fig. S12e–g). These results suggested that OXT treatment in adulthood could only temporarily rescue social-behavioral dysfunction in BWT mice.

### Early administration of OXT was sufficient to recover social discrimination deficit in Fmr1 knockout mice

**Fig. 6:**
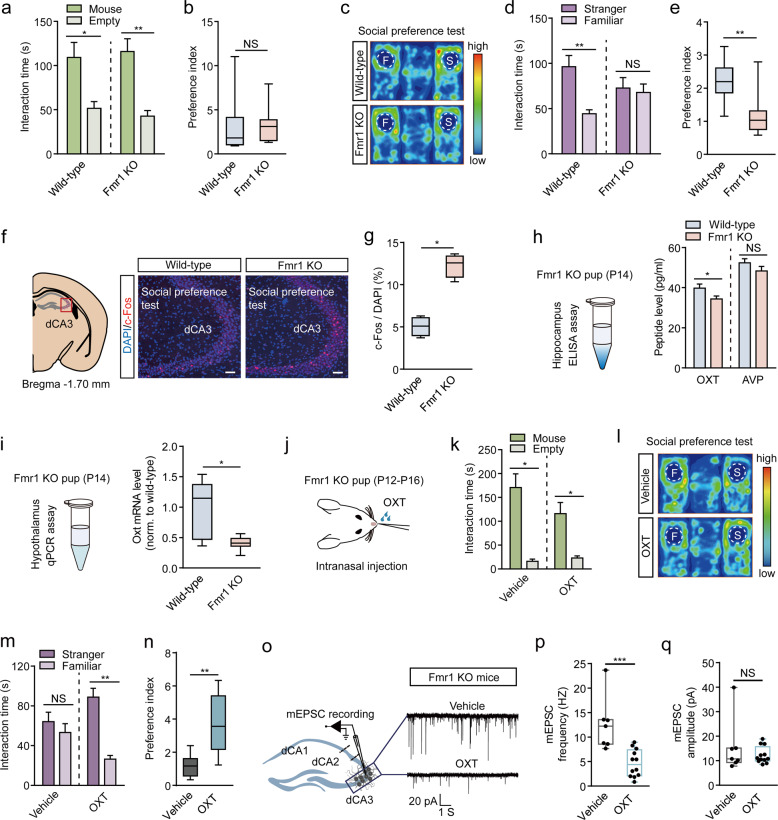
Early life oxytocin treatment rescues social discrimination deficit in adult Fmr1 KO mice. **a**, **b** Sociability test. Time spent (**a**, Wild-type: *n* = 8 mice, *P* = 0.0285, paired *t*-test; Fmr1 KO: *n* = 10 mice, *P* = 0.0020, Wilcoxon matched-pairs signed-rank test) and preference (**b**, *P* = 0.9484, unpaired *t*-test) for sniffing the mouse chamber or the empty chamber. **c**–**e** Social preference test. **c** Representative heatmaps. **d**, **e** Social preference in the three-chamber test showing time spent (**d**, wild-type: *n* = 8 mice, *P* = 0.0078, Wilcoxon matched-pairs signed-rank test; Fmr1 KO: *n* = 10 mice, *P* = 0.6089, paired *t*-test) and preference (**e**, *P* = 0.0044, Mann–Whitney *U* test.) for interacting with a stranger mouse versus a familiar mouse. **f**, **g** c-Fos analysis for wild-type and Fmr1 KO mice. **f** Example images of c-Fos-positive neurons in the dCA3. Scale bar, 50 μm. **g** Number of c-Fos-positive neurons for wild-type and Fmr1 KO mice (Each group, *n* = 4 mice, *P* = 0.0286, Mann–Whitney test). **h** Left, diagram shows the peptide ELISA assay. Right, OXT and AVP peptide levels in the hippocampus for wild-type and Fmr1 KO mice. Wild-type: *n* = 16 mice; Fmr1 KO: *n* = 18 mice. OXT: *P* = 0.0377; AVP: *P* = 0.1946; unpaired *t*-test. **i** Oxytocin mRNA level in the hypothalamus in wild-type and Fmr1 KO mice (wild-type: *n* = 8 mice; Fmr1 KO: *n* = 15 mice; *P* = 0.0130; Mann–Whitney *U* test). **j** Schematic shows intranasal OXT treatment. **k** Sociability test. Vehicle: *n* = 6 mice, *P* = 0.0313; OXT: *n* = 7 mice, *P* = 0.0156; Wilcoxon matched-pairs signed-rank test. **i**–**n** Social preference test. **i** Representative heatmaps. Time spent (**m**, Vehicle: *P* = 0.6784, OXT: *P* = 0.0049; paired *t*-test) and preference (**n**, *P* = 0.0093, unpaired *t*-test) for interacting with a stranger mouse versus a familiar mouse. **o** Representative spontaneous mEPSC recordings in dCA3 cells from vehicle (upper) and OXT (lower) for Fmr1 KO mice. **p**, **q** mEPSC frequencies (**p**, *P* = 0.0006, unpaired *t*-test) and mEPSC amplitudes (**q**, *P* = 0.8666, Mann–Whitney *U* test) in dCA3 (vehicle, *n* = 7 neurons from 3 mice; OXT, *n* = 12 neurons from 3 mice). *P < 0.05; **P < 0.01; ***P < 0.001; NS, not significant. Data presented as mean ± SEM.

We next wanted to see if the over-activation of dCA3 is relevant to social behavior abnormalities in mouse models for human diseases, such as in Fmr1 KO mice that represent a mouse model for FXS. In line with a previous study [44], in the sociability test, both Fmr1 KO mice and control littermates presented similar and significantly increased interest in the chamber containing a mouse compared to the empty chamber (Fig. 6a, b). However, unlike control mice, the Fmr1 KO mice did not exhibit any increased interaction preference toward the novel mouse over the familiar one in the three-chamber social preference test (Fig. 6c–e). This suggested that social discrimination was impaired in Fmr1 KO mice. Next, we performed *c-Fos* immunostaining 1.5 h after the social preference test, and found that Fmr1 KO mice displayed significantly more *c-Fos*-positive cells in the dCA3 area compared to the control groups (Fig. 6f, g). To further validate the relationship between over-activation of dCA3 and abnormal social discrimination in Fmr1 KO mice, we attempted to suppress dCA3 activity via chemogenetics. We first expressed hM4Di by injection of the AAV-CaMKIIα-hM4Di-eGFP virus and injection of AAV-CaMKIIα-eGFP virus as control into bilateral dCA3 of Fmr1 KO mice and then allowed the mice to recover for 3 weeks. We then performed a three-chamber social test 30 min after the mice had received CNO or saline treatments (Supplementary Fig. S13a–e). Compared to those receiving saline treatment, hM4Di-expressed Fmr1 KO mice receiving CNO treatment did not change their preference toward a mouse over an empty chamber (Supplementary Fig. S13d, e), but recovered their interest and spent significantly more time exploring the unfamiliar mouse than the familiar mouse during the social preference test (Supplementary Fig. S13f–i). CNO treatment in eGFP expressing Fmr1 KO mice failed to rescue the social discrimination deficit (Supplementary Fig. S13g–i). Similar to the results of the BWT mice, the social discrimination deficit in Fmr1 KO mice was therefore attributed to over-activation of the dCA3 area.

To investigate the possible role of OXT signaling in the social discrimination deficit of Fmr1 KO mice, we collected hippocampal samples from P14 Fmr1 KO and littermate control mice. ELISA assay results consistently revealed significantly reduced hippocampal OXT, but not AVP levels in Fmr1 KO mice (Fig. 6h). We also observed a remarkable reduction of OXT mRNA level in the hypothalamus using qRT-PCR for P14 Fmr1 KO mice (Fig. 6i). In addition, the OXT-positive cells in the PVN, but not in the SON, were also significantly decreased in the Fmr1 KO mice when compared to littermate controls (Supplementary Fig. S14). These results strongly suggest that the OXT might be involved in social behavior dysfunction in Fmr1 KO mice.

Next, we wanted to investigate whether the intranasal application of OXT during early life was sufficient to rescue the social discrimination deficit in Fmr1 KO mice in adulthood. We first performed intranasal administration of OXT, and ACSF as control, twice a day from P12–16 in Fmr1 KO mice and then waited until the mice were 2 months old (Fig. 6j). Interestingly, we found that early life intranasal OXT treatment was sufficient to rescue the social discrimination deficit in Fmr1 KO mice, whereas those mutants having received intranasal ACSF treatment did not show this rescue (Fig. 6k–n). Fmr1 KO mice receiving early life intranasal OXT treatment, but not ACSF treatment, spent more time interacting with a novel mouse over a familiar one in the three-chamber social preference test. The reduced dCA3 spontaneous mEPSC frequency was also associated with the recovery of social discrimination deficit in Fmr1 KO mice having received early life intranasal OXT treatment (Fig. 6o–q). We also explored the effects of adult OXT treatment in Fmr1 KO mice. Similar to BWT mice, social-behavioral dysfunction in Fmr1 mice could only be rescued 30 min after adult OXT treatment, but not after 24 h (Supplementary Fig. S15).

Lastly, we found early life intracerebroventricular OXT treatment could also rescue the social discrimination deficit of Fmr1 KO mice, whereas those mutants having received ACSF treatment showed no such rescue (Supplementary Fig. S16a–e). In addition, the rescued social discrimination deficit was associated with a reduced dCA3 spontaneous mEPSC frequency, but not amplitude, in adult Fmr1 KO mice having undergone early life intracerebroventricular OXT treatment (Supplementary Fig. S16f–h).

## Discussion

Although the phenomena of intimate interconnections between early life tactile experience and social behavior in adulthood have been under consideration for decades, the underlying mechanisms remain unclear. In our study, we observed that only a short period of whisker trimming during early life could have a profound impact on the adult mouse's social behaviors, with a specific impairment upon social discrimination. Whiskers are one of the most important tactile organs in rodents and the whisker sensory experience during a critical window in early development seems to serve a vital role in guiding the pruning of redundant synapses [6, 45]. Lack of whisker sensory experience may cause the reduction of neural activity-dependent synthesis and the release of neurotrophic factors or neuropeptides, and thereby affecting the developmental synaptic maturation and circuit formation that is crucial for proper behaviors, including social behavior. In line with this opinion, one recent study found that unimodal sensory deprivation, such as dark rearing or whisker deprivation during early life, could cause extensive alterations among various sensory cortical areas through the modulation of OXT-mediated signaling [19]. Consistent with this, our results reveal that early life whisker trimming is correlated to a reduction of the OXT level in the hippocampus, as well as dCA3 over-activation. However, we could not rule out a possibility of altered Oxtr in BWT and Fmr1 KO mice, as one recent study suggests that a delay in GABAergic development associated with an increase in the quantity of Oxtrs and of somatostatin interneurons in both DG and CA2/CA3 regions of hippocampus contributes to social memory deficit in Magel2 KO mice [21]. Therefore, further studies are needed to address whether the function of Oxtr is also altered in BWT and Fmr1 KO mice. Taken together, the impairments of early postnatal oxytonergic pathways subsequently caused the mice to display impaired social function. These studies indicated that more than just a social hormone, OXT signaling plays an important role in precise neural connectivity formation, in particular for circuits related to social behavior. Surprisingly, the social discrimination deficit in Fmr1 KO mice was also attributed to the over-activation of the dCA3 area, and was rescued by OXT signaling compensation. In line with our results, recent studies showed that OXT-positive cells were reduced [46] and the spontaneous mEPSC in dCA3 was enhanced in Fmr1 KO mice [47]. Despite dCA3 hyperactivity seeming to be a common feature between BWT and Fmr1 KO mice, it will be of great interest to test whether over-activation of dCA3 due to neonatal loss-of-function of OXT signaling represents a conserved and universal rule underlying social behavior dysfunction.

One caveat is how reduced OXT levels enhance the neural activity in dCA3. The difference in OXT levels between BWT and control mice appears to be slight, with a significant reduction observed in BWT mice. It is possible that during early postnatal development, dendritic branching and synapse formation in the dCA3 are very sensitive to changes in OXT levels. Previous studies have suggested that whisker trimming during early life significantly affects neural activity in somatosensory pathways, such as impairing synaptic refinement in the ventral posteromedial nucleus (VPm) of the thalamus or reducing synaptic function in somatosensory cortex [6, 48]. Although using the retrograde virus tracing technique we failed to observe any direct projections from the somatosensory system such as from the VPm or the somatosensory cortex to the dCA3, the enhanced dCA3 neural activity still could be a homeostatic compensation for reduced activity in other brain regions. Alternatively, the enhanced dCA3 neural activity could be a result of reduced hippocampal OXT levels during early postnatal development. Several lines of evidence support this latter notion. Firstly, we found that mice with neonatal Oxtr knockdown in dCA3 have impairments in social preference test, and could not be rescued by concurrently early life OXT treatment. Further study revealed that mEPSC frequency was also increased in Oxtr knockdown mice. This result suggested that OXT may act on Oxtr in dCA3 to regulate social behavior. Secondly, a study by Silvia Ripamonti et al. demonstrated that transient OXT treatment reduces dendrite branching and the functioning of hippocampus glutamatergic neurons via Gq/11-coupled Oxtrs [49]. Thirdly, previous studies have suggested that the impairment of oxytocinergic signaling pathways abolishes the perinatal excitatory-to-inhibitory shift response of neurons to GABA, thereby enhancing the spontaneous excitatory neural activity of the hippocampal CA3 area to result in social behavior dysfunction in different ASD rodent models, including Fmr1 KO mice [47].

It is important to note that in the current study we did not test female mice. This is due to several reasons. Firstly, as suggested by previous studies, there is a less obvious preference to interact with a novel female compared with a familiar one in the social discrimination test for female mice [21]. Secondly, males have a much higher prevalence of autism than females, for example, FXS. Thirdly, in experiments that span several days, females are more influenced by fluctuating hormone levels than males, thus causing large variations. Nevertheless, female mice could and maybe should be tested in future studies with appropriate behavioral assays.

It seems that the hyperactivity of dCA3 acts as internal noise to interfere with social memory retrieval. This is demonstrated by the dramatic increase in activity in a large population of neurons during the social preference test, when interacting with both the familiar and strange mice, but not during the sociability test in BWT mice. Although we have shown that vCA1 might be the downstream target of dCA3 in social behavior circuitry, it will be of great interest to further explore how over-activation of the dCA3 is coordinated with known social memory-related nuclei including ventral CA1 [30] and CA2 [31], to impair social behavior.

Overall, our study uncovered a novel mechanism to explain why early life tactile experiences are important for the building of appropriate social behavior. Our results support the view that increased hippocampal activation is a dysfunctional condition [50] and we propose that targeting excess hippocampal dCA3 activity via intranasal administration of OXT early postnatal life may have therapeutic potential in the prevention of social-behavioral deficit-related disorders.

## Supplementary information

Supplementary Materials

Supplementary movie 1

Supplementary movie 2

Supplementary movie 3

Supplementary movie 4

## References

[1] Chedotal A, Richards LJ (2010). Wiring the brain: the biology of neuronal guidance. Cold Spring Harb Perspect Biol.

[2] Pan Y, Monje M (2020). Activity shapes neural circuit form and function: a historical perspective. J Neurosci.

[3] Hua JY, Smith SJ (2004). Neural activity and the dynamics of central nervous system development. Nat Neurosci.

[4] Katz LC, Shatz CJ (1996). Synaptic activity and the construction of cortical circuits. Science.

[5] LeBlanc JJ, Fagiolini M (2011). Autism: a “critical period” disorder?. Neural Plasticity.

[6] Wang H, Zhang ZW (2008). A critical window for experience-dependent plasticity at whisker sensory relay synapse in the thalamus. J Neurosci.

[7] Simons DJ, Land PW (1987). Early experience of tactile stimulation influences organization of somatic sensory cortex. Nature.

[8] Hong YK, Chen C (2011). Wiring and rewiring of the retinogeniculate synapse. Curr Opin Neurobiol.

[9] Hooks BM, Chen C (2006). Distinct roles for spontaneous and visual activity in remodeling of the retinogeniculate synapse. Neuron.

[10] Kandler K, Clause A, Noh J (2009). Tonotopic reorganization of developing auditory brainstem circuits. Nat Neurosci.

[11] Pomeroy SL, LaMantia AS, Purves D (1990). Postnatal construction of neural circuitry in the mouse olfactory bulb. J Neurosci.

[12] McGlone F, Wessberg J, Olausson H (2014). Discriminative and affective touch: sensing and feeling. Neuron.

[13] Meaney MJ (2001). Maternal care, gene expression, and the transmission of individual differences in stress reactivity across generations. Annu Rev Neurosci.

[14] Sale A, Berardi N, Maffei L (2014). Environment and brain plasticity: towards an endogenous pharmacotherapy. Physiological Rev.

[15] Lee LJ, Chen WJ, Chuang YW, Wang YC (2009). Neonatal whisker trimming causes long-lasting changes in structure and function of the somatosensory system. Exp Neurol.

[16] Papaioannou S, Brigham L, Krieger P (2013). Sensory deprivation during early development causes an increased exploratory behavior in a whisker-dependent decision task. Brain Behav.

[17] Soumiya H, Godai A, Araiso H, Mori S, Furukawa S, Fukumitsu H (2016). Neonatal whisker trimming impairs fear/anxiety-related emotional systems of the amygdala and social behaviors in adult mice. PloS One.

[18] Bureau I, Shepherd GM, Svoboda K (2008). Circuit and plasticity defects in the developing somatosensory cortex of FMR1 knock-out mice. J Neurosci.

[19] Zheng JJ, Li SJ, Zhang XD, Miao WY, Zhang D, Yao H (2014). Oxytocin mediates early experience-dependent cross-modal plasticity in the sensory cortices. Nat Neurosci.

[20] Meziane H, Schaller F, Bauer S, Villard C, Matarazzo V, Riet F (2015). An early postnatal oxytocin treatment prevents social and learning deficits in adult mice deficient for Magel2, a gene involved in Prader-Willi syndrome and autism. Biol Psychiatry.

[21] Bertoni A, Schaller F, Tyzio R, Gaillard S, Santini F, Xolin M, et al. Oxytocin administration in neonates shapes hippocampal circuitry and restores social behavior in a mouse model of autism. Mol Psychiatry. 2021;26:7582–95. 10.1038/s41380-021-01227-6 PMC8872977 34290367

[22] Froemke RC, Young LJ. Oxytocin, neural plasticity, and social behavior. Annu Rev Neurosci. 2021;44:359–81. 10.1146/annurev-neuro-102320-102847 PMC8604207 33823654

[23] Tang Y, Benusiglio D, Lefevre A, Hilfiger L, Althammer F, Bludau A (2020). Social touch promotes interfemale communication via activation of parvocellular oxytocin neurons. Nat Neurosci.

[24] Francis SM, Sagar A, Levin-Decanini T, Liu W, Carter CS, Jacob S (2014). Oxytocin and vasopressin systems in genetic syndromes and neurodevelopmental disorders. Brain Res.

[25] Andari E, Duhamel JR, Zalla T, Herbrecht E, Leboyer M, Sirigu A (2010). Promoting social behavior with oxytocin in high-functioning autism spectrum disorders. Proc Natl Acad Sci USA.

[26] Parker KJ, Oztan O, Libove RA, Sumiyoshi RD, Jackson LP, Karhson DS (2017). Intranasal oxytocin treatment for social deficits and biomarkers of response in children with autism. Proc Natl Acad Sci USA.

[27] Harony-Nicolas H, Kay M, du Hoffmann J, Klein ME, Bozdagi-Gunal O, Riad M, et al. Oxytocin improves behavioral and electrophysiological deficits in a novel Shank3-deficient rat. eLife. 2017;6:e18904. 10.7554/eLife.18904 PMC5283828 28139198

[28] Resendez SL, Namboodiri VMK, Otis JM, Eckman LEH, Rodriguez-Romaguera J, Ung RL (2020). Social stimuli induce activation of oxytocin neurons within the paraventricular nucleus of the hypothalamus to promote social behavior in male mice. J Neurosci.

[29] Penagarikano O, Lazaro MT, Lu XH, Gordon A, Dong H, Lam HA (2015). Exogenous and evoked oxytocin restores social behavior in the Cntnap2 mouse model of autism. Sci Transl Med.

[30] Okuyama T, Kitamura T, Roy DS, Itohara S, Tonegawa S (2016). Ventral CA1 neurons store social memory. Science.

[31] Hitti FL, Siegelbaum SA (2014). The hippocampal CA2 region is essential for social memory. Nature.

[32] Gunaydin LA, Grosenick L, Finkelstein JC, Kauvar IV, Fenno IE, Adhikari A (2014). Natural neural projection dynamics underlying social behavior. Cell.

[33] Tirko NN, Eyring KW, Carcea I, Mitre M, Chao MV, Froemke RC (2018). Oxytocin transforms firing mode of CA2 hippocampal. Neurons Neuron.

[34] Lin YT, Hsu KS (2018). Oxytocin receptor signaling in the hippocampus: role in regulating neuronal excitability, network oscillatory activity, synaptic plasticity and social memory. Prog Neurobiol.

[35] Yang J, Yang H, Liu Y, Li X, Qin L, Lou H (2016). Astrocytes contribute to synapse elimination via type 2 inositol 1,4,5-trisphosphate receptor-dependent release of ATP. eLife.

[36] Yang M, Crawley JN. Simple behavioral assessment of mouse olfaction. Curr Protoc Neurosci. 2009;Chapter 8:Unit 8.24. 10.1002/0471142301.ns0824s48 PMC2753229 19575474

[37] DeVito LM, Konigsberg R, Lykken C, Sauvage M, Young WS, Eichenbaum H (2009). Vasopressin 1b receptor knock-out impairs memory for temporal order. J Neurosci.

[38] Brennan PA, Zufall F (2006). Pheromonal communication in vertebrates. Nature.

[39] Miyashita T, Shao YR, Chung J, Pourzia O, Feldman DE (2013). Long-term channelrhodopsin-2 (ChR2) expression can induce abnormal axonal morphology and targeting in cerebral cortex. Front Neural Circuits.

[40] Fakira AK, Massaly N, Cohensedgh O, Berman A, Moron JA (2016). Morphine-associated contextual cues induce structural plasticity in hippocampal CA1 pyramidal neurons. Neuropsychopharmacology.

[41] Donaldson ZR, Young LJ (2008). Oxytocin, vasopressin, and the neurogenetics of sociality. Science.

[42] Modi ME, Young LJ (2012). The oxytocin system in drug discovery for autism: animal models and novel therapeutic strategies. Hormones Behav.

[43] Hofmann J, Huber C, Novak B, Schreckenbach M, Schubert CF, Touma C (2021). Oxytocin receptor is a potential biomarker of the hyporesponsive HPA axis subtype of PTSD and might be modulated by HPA axis reactivity traits in humans and mice. Psychoneuroendocrinology.

[44] Yau SY, Chiu C, Vetrici M, Christie BR (2016). Chronic minocycline treatment improves social recognition memory in adult male Fmr1 knockout mice. Behav Brain Res.

[45] Pan L, Yang J, Yang Q, Wang X, Zhu L, Liu Y (2017). A critical period for the rapid modification of synaptic properties at the VPm relay synapse. Front Mol Neurosci.

[46] Carter CS, Grippo AJ, Pournajafi-Nazarloo H, Ruscio MG, Porges SW (2008). Oxytocin, vasopressin and sociality. Prog Brain Res.

[47] Tyzio R, Nardou R, Ferrari DC, Tsintsadze T, Shahrokhi A, Eftekhari S (2014). Oxytocin-mediated GABA inhibition during delivery attenuates autism pathogenesis in rodent offspring. Science.

[48] Wen JA, Barth AL (2011). Input-specific critical periods for experience-dependent plasticity in layer 2/3 pyramidal neurons. J Neurosci.

[49] Ripamonti S, Ambrozkiewicz MC, Guzzi F, Gravati M, Biella G, Bormuth I, et al. Transient oxytocin signaling primes the development and function of excitatory hippocampal neurons. eLife. 2017;6:e22466. 10.7554/eLife.22466 PMC5323041 28231043

[50] Bakker A, Krauss GL, Albert MS, Speck CL, Jones LR, Stark CE (2012). Reduction of hippocampal hyperactivity improves cognition in amnestic mild cognitive impairment. Neuron.

