# Supplementary material for: A short period of early life oxytocin treatment rescues social behavior dysfunction via suppression of hippocampal hyperactivity in male mice
Source: Mol Psychiatry. 2022 Jul 15;27(10):4157–71. doi: 10.1038/s41380-022-01692-7 (PMC9718675; doi:10.1038/s41380-022-01692-7)
Supplement: Supplementary file 1 — Supplementary Materials [file 41380_2022_1692_MOESM1_ESM.docx]

**Supplementary Figures**

**
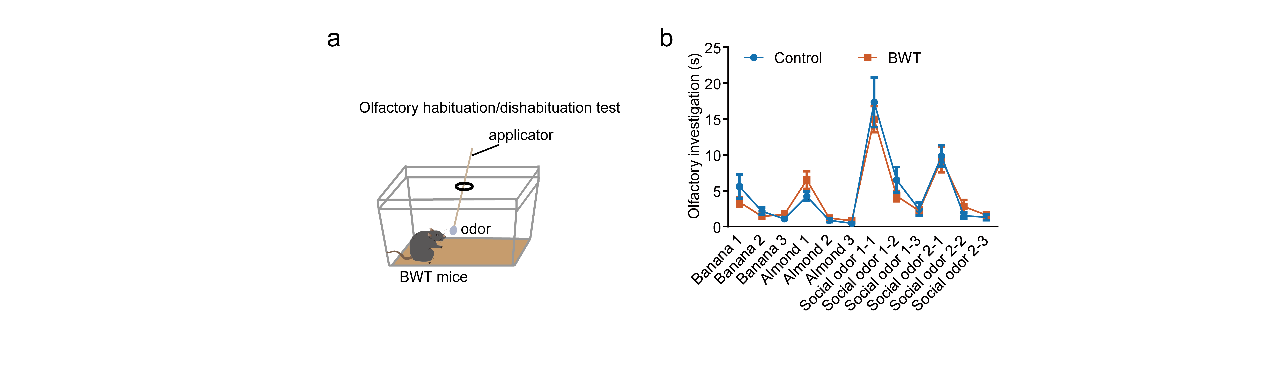
**

**Figure S1. The ability to detect or discriminate non-social or social odors remain unaltered in BWT mice.**

**a**, Schematic shows experimental protocol.

**b**, There were no significant differences between the performance of groups in the olfactory habituation/dishabituation task. Control: n = 9 mice; BWT: n = 9 mice. P = 0.6505, Two-way ANOVA.

Data presented as mean ± SEM.

**
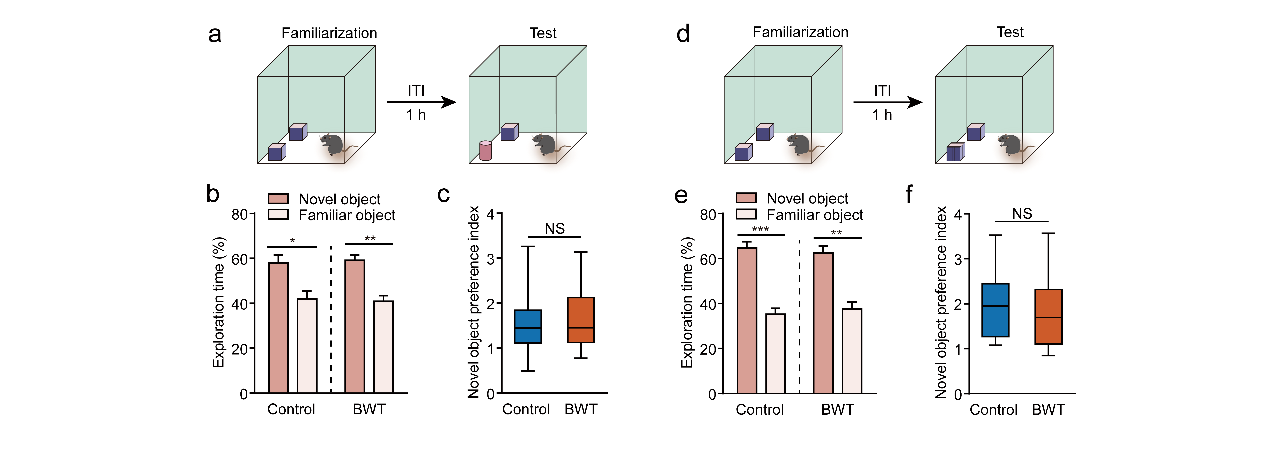
**

**Figure S2. Novel object recognition** **and object-texture-recognition are not affected in BWT mice.**

**a**, Diagram of the experimental design for the novel-object-recognition task.

**b**,**c**, Discrimination ratio (**b**, Control: n = 12 mice, P = 0.0431; BWT: n = 16 mice, P = 0.0022; paired t test) and preference (**c**, P = 0.9744, unpaired t test) of the time mice spent in exploring the novel or familiar object.

**d**, Diagram of the experimental design for the object-texture-recognition task.

**e**,**f**, Discrimination ratio (**e**, Control: n = 10 mice, P = 0.0004; BWT, n = 11 mice, P = 0.0023; Paired t test) and preference (**f**, P = 0.7061, unpaired t test) of the time mice spent in exploring the novel or familiar objects.

NS, not significant. Data presented as mean ± SEM.


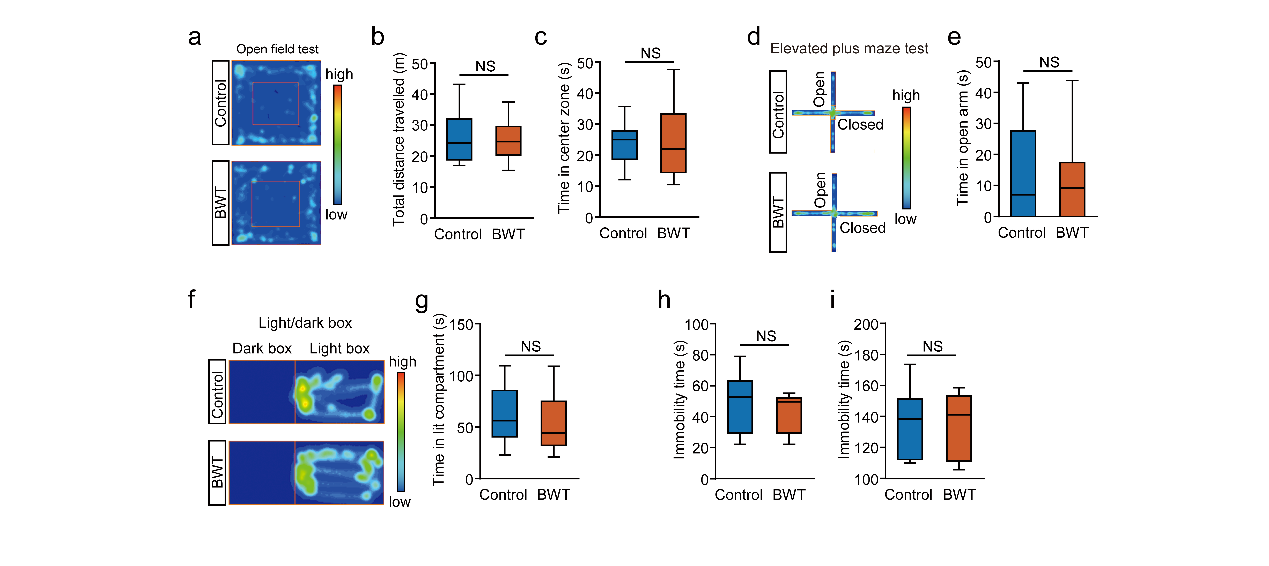


**Figure S3. No anxiety-like or depression-like behaviors were observed in BWT mice.**

**a**-**c**, Open field test. **a,** Representative animal heat maps. **b**, **c**, There was no significant difference between control and BWT mice in the total distance traveled (**b**, Control: n = 18 mice; BWT: n = 14 mice; P = 0.7091, unpaired t test) or time spent (**c**, P = 0.8883, Mann-Whitney U test) in the center zone in the open field test.

**d**, **e**, Elevated plus maze (EPM) test. **d**, Representative heat maps of EPM test. **e**, Time spent in open arms did not differ significantly between the groups. Control: n = 16 mice; BWT: n = 16 mice; P = 0.9172, Mann-Whitney U test.

**f**, **g**, Light/dark box test. **f**, Representative heat maps of light/dark box test. **g**, Time spent in the lit compartment did not significantly differ between the groups. Control: n = 18 mice; BWT: n = 18 mice; P = 0.4003, unpaired t test.

**h**, **i**, The immobility time in the forced swimming (**h**, Control: n = 6 mice; BWT, n = 6 mice; P = 0.4719 , Mann-Whitney U test）or in the tail suspension (**i**, Control: n = 6 mice; BWT, n = 6 mice; P = 0.7673, unpaired t test) tests did not significantly differ between the groups.

NS, not significant. Error bars indicate SEM.

**
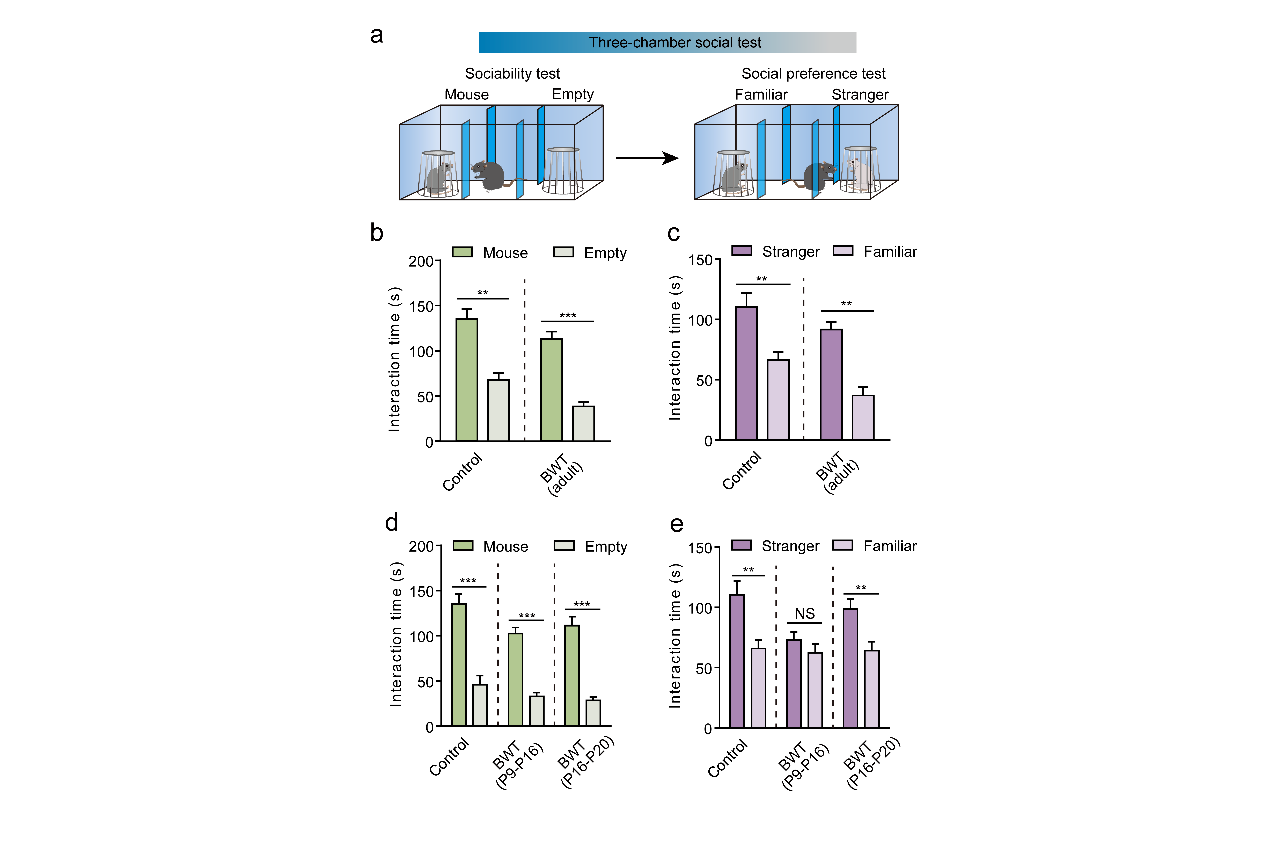
**

**Figure S4. Social function of adult mice underwent whisker trimming during different time periods.**

**a**, Diagram showing the three chamber test.

**b**, Sociability test of mice underwent 5 days whisker trimming in adult. Time spent (**c**, Control: n = 11 mice, P = 0.0002; BWT: n = 6 mice, P = 0.0001; paired t test) for sniffing the mouse chamber or the empty chamber.

**c**, Social discrimination in the three-chamber test showing time spent (**f**, Control: P = 0.0011; BWT: P = 0.0015; paired t test) for interacting with a stranger mouse versus a familiar mouse.

**d**, Sociability test. Control: n = 11 mice, P = 0.0002; BWT (P9-P16): n = 10 mice, P < 0.0001; BWT (P16-P20): n = 10 mice, P < 0.0001; paired t test.

**e**, Social preference test. Control: P = 0.0011; BWT (P9-P16): P = 0.1716; BWT (P16-P20): P = 0.0010; paired t test.

**P < 0.01; ***P < 0.001; NS, not significant. Data presented as mean ± SEM.


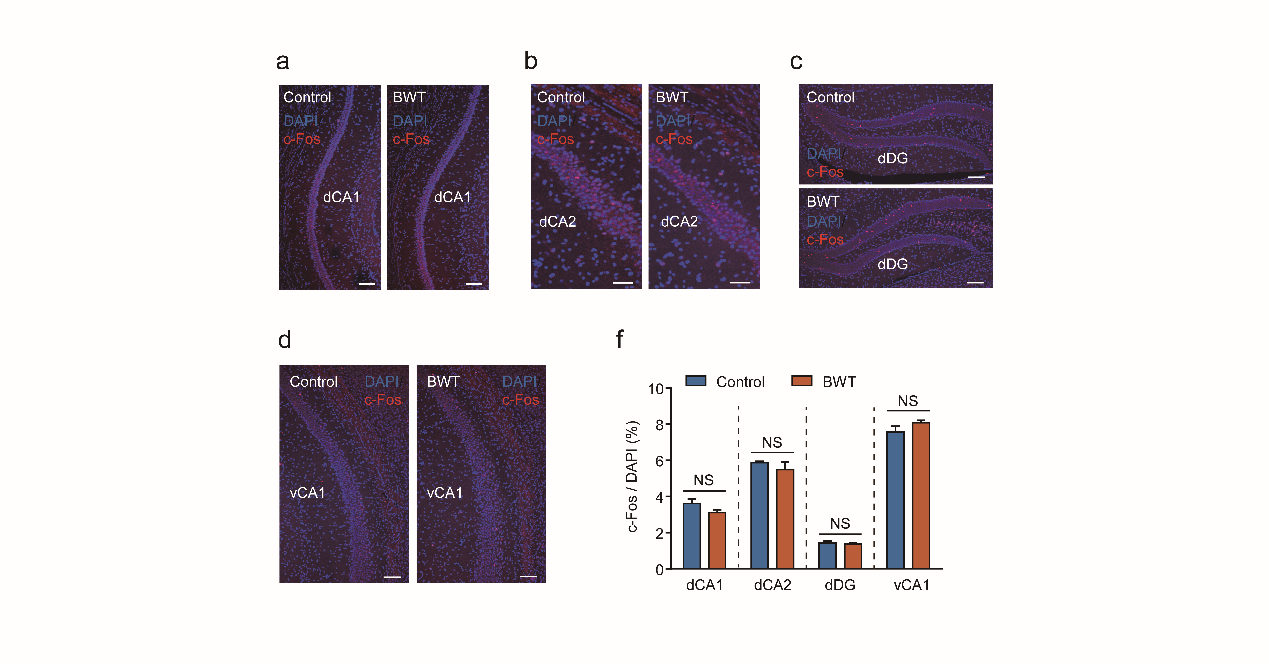


**Figure S5. c-Fos expression in the subregions of hippocampus after social preference test.**

**a**-**d**, Representative images of c-Fos expression in the dCA1 (**a**), dCA2 (**b**), dDG (**c**) and vCA1 (**d**) from control and BWT groups 1.5 h after the social preference test. **a**, **c** and **d**, scale bar indicates 100 μm; **b**, scale bar indicates 50 μm.

**e**, Quantification of the numbers of cells expressing c-Fos in the dCA1 (n = 4 mice, each group; P = 0.2000), dCA2 (n = 4 mice, each group; P > 0.9999), dDG (n = 4 mice, each group; P = 0.6571) and vCA1 (n = 4 mice, each group; P = 0.2286). Mann Whitney test.

NS, not significant. Error bars indicate SEM.

**
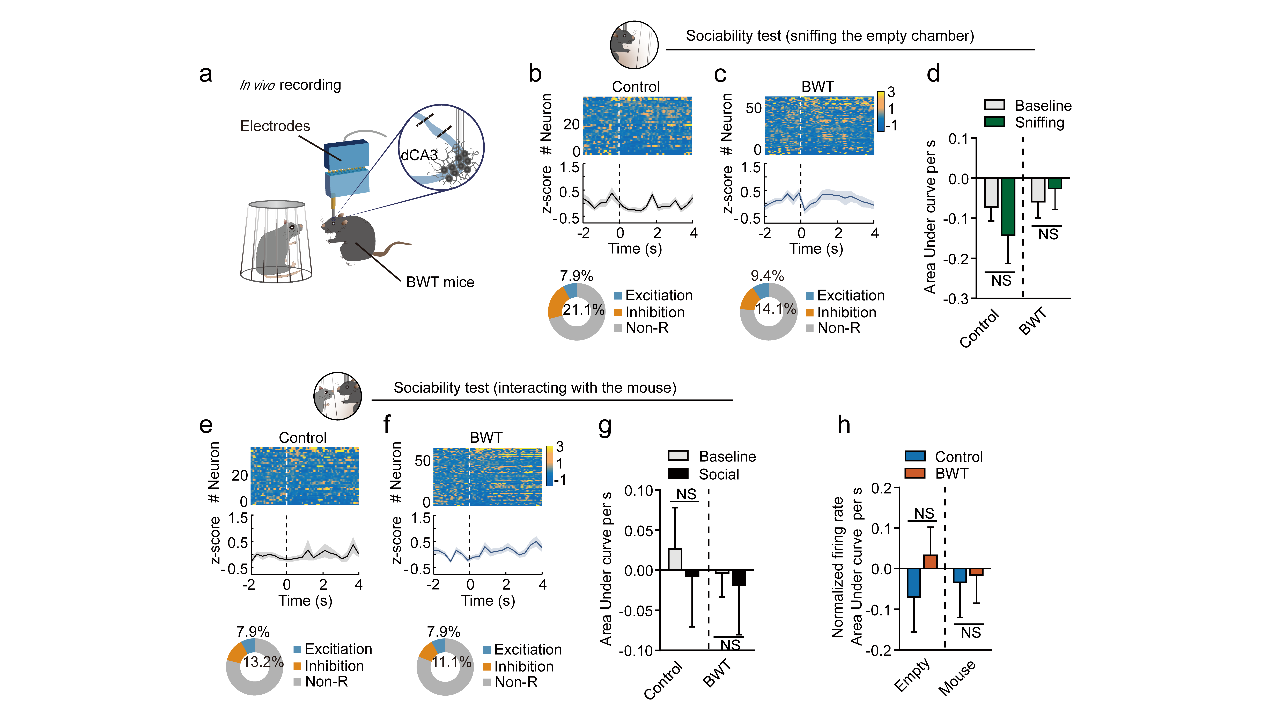
**

**Figure S6.** **Whisker trimming through P12-16 did not change the activity of dCA3 neurons during sociability testing.**

**a**, The diagram shows the tetrode recording strategy in the dCA3. Control: n = 38 neurons from 5 mice; BWT: n = 63 neurons from 8 mice.

**b**-**d**, Tetrode recording of sniffing the empty chamber during sociability test. **b**,**c**, Heatmaps of normalized z-scored activity (**b** and **c** upper) and average activity (**b** and **c** middle) of the recorded neurons. Relative proportions of neurons deemed excited (blue), inhibited (yellow), or unaffected (gray) (**b** and **c** lower). T = 0 s denotes the time when sniffing starts. **d**, Mean population activity before and during (control: P = 0.3209; BWT: P = 0.4114; Wilcoxon matched-pairs signed rank test) sniffing the empty chamber.

**e**-**g**, Tetrode recording of interaction with the mouse. **e**, **f**, Heatmaps of normalized z-scored activity (**e** and **f** upper) and average activity (**e** and **f** middle) of the recorded neurons. Relative proportions of neurons deemed excited (blue), inhibited (yellow), or unaffected (gray) (**e** and **f** lower). T = 0 s denotes the time when social interaction starts. **g,** Mean population activity before and during (control: P = 0.8298; BWT: P = 0.2856; Wilcoxon matched-pairs signed rank test) social interaction.

**h**, The normalized firing rate of BWT mice neurons did not show any changes during the sociability test. Empty: P = 0.1694; Mouse: P = 0.5545. Mann-Whitney U test.

NS, not significant. Error bars indicate SEM.

**
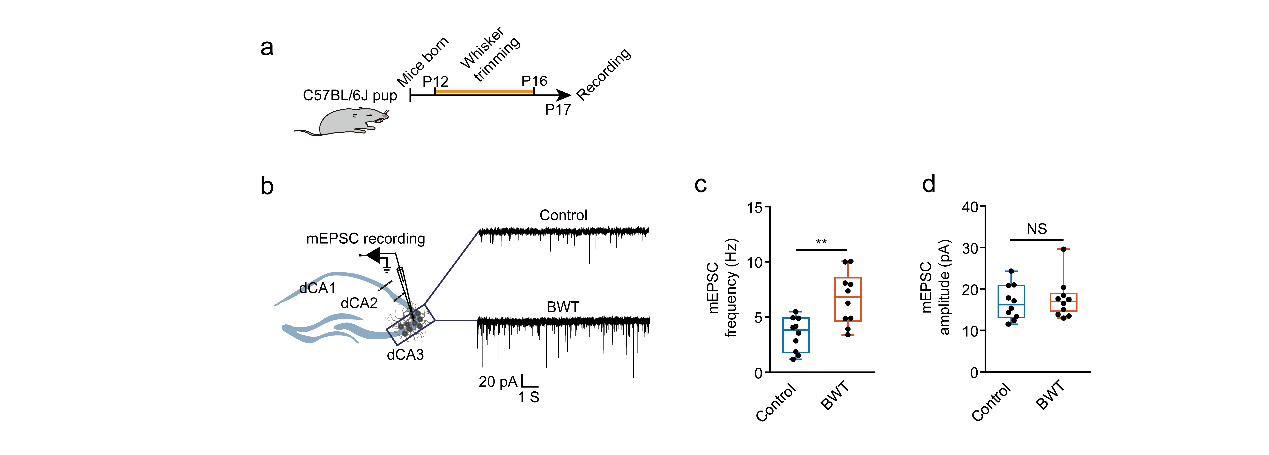
Figure S7. Whikser trimming during P12-16 affects dCA3 neural activity at P17**

**a**, Experimental strategy diagram. C57BL/6J pups with bilateral whisker trimming experience during P12-P16 were performed whole cell recording at P17.

**b**, Representative mEPSC recordings from control mice (upper) and BWT mice (lower).

**c**, **d**, mEPSC frequencies (**c**, P = 0.0021, unpaired t test) and mEPSC amplitudes (**d**, P = 0.7176, unpaired t test). Control, n = 10 neurons from 3 mice; BWT, n = 10 neurons from 3 mice.

**P < 0.01; NS, not significant. Data presented as mean ± SEM.


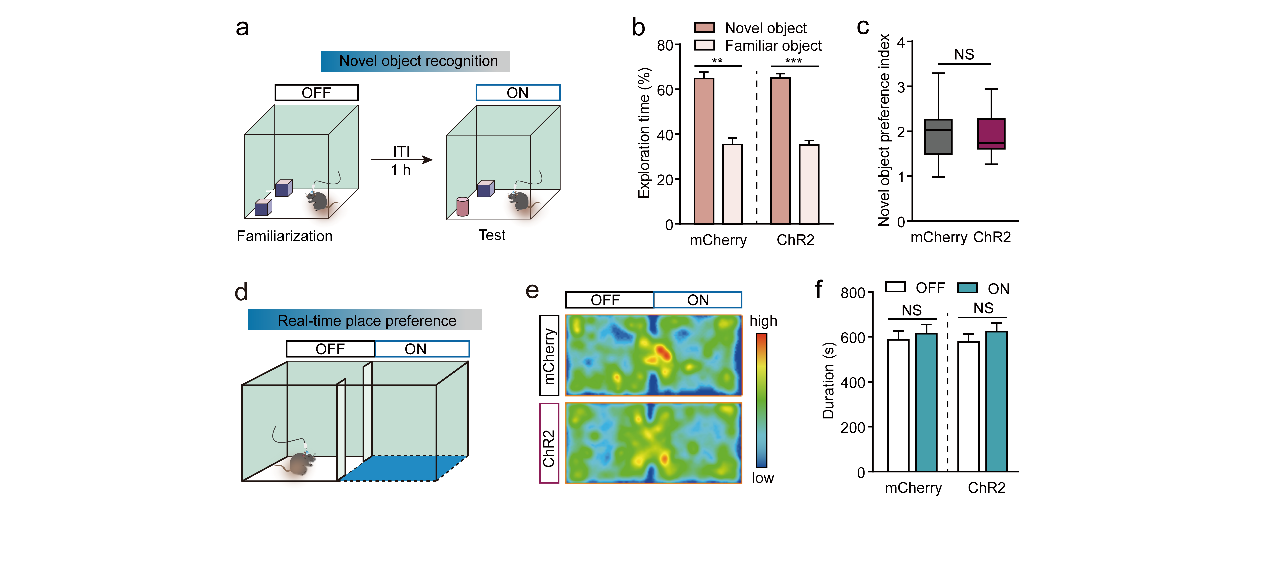


**Figure S8. Optogenetic activation of hippocampus in wild type mice does not affect novel object recognition, nor induce place pereference.**

**a**, Behavior paradigm. The optical stimulation of dCA3 neurons was administered in the test phase.

**b**, Both ChR2 (n = 8 mice, P = 0.0002, paired t test) and mCherry (n = 9 mice, P = 0.0078, Wilcoxon matched-pairs signed rank test) groups spent more time exploring the novel object than the familiar object.

**c**, The novel object preference index did not display significant differences between the mCherry and ChR2 groups. P = 0.8801, unpaired t test.

**d**, Schematic shows the real-time place preference test.

**e**, Representative heat maps.

**f**, Time spent in each chamber during the test. mCherry: n = 8 mice, P = 0.7405; ChR2: n = 10 mice, P = 0.5324; paired t test.

**P < 0.01; ***P < 0.001; NS, not significant. Error bars indicate SEM.

**
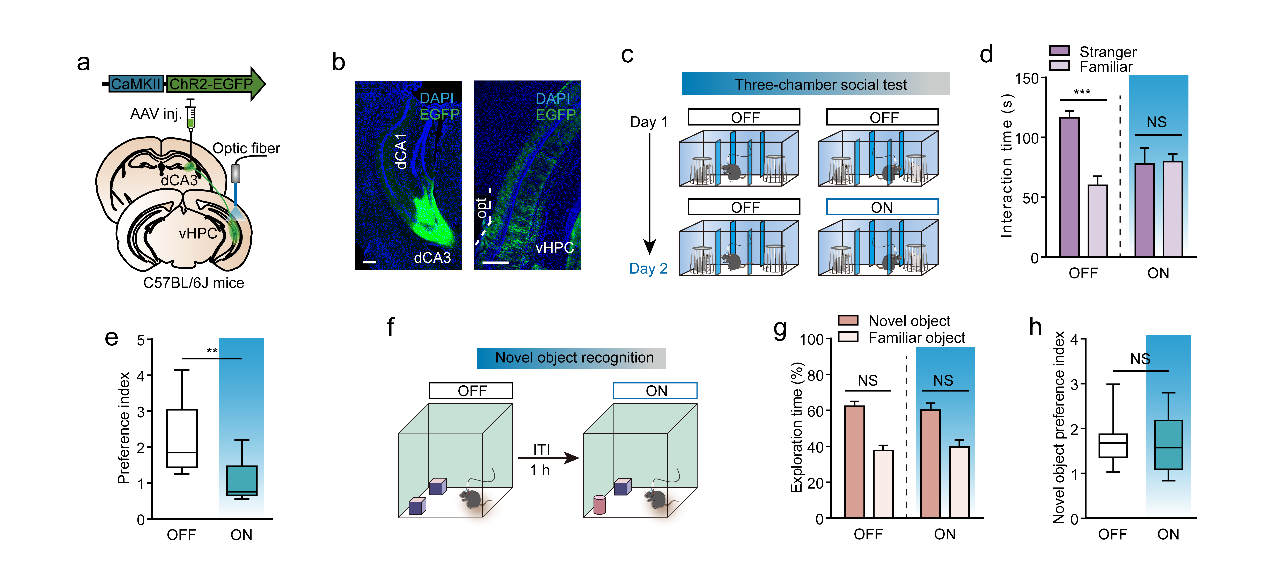
Figure S9. Optogenetic activation of dCA3-vCA1 pathway induced social discrimination deficit.**

**a**, C57BL/6J mice injected with AAV-CaMKIIα-hChR2-mCherry into the hippocampal dCA3 and the optical fiber implanted above vCA1.

**b**, Image of the dCA3 (left) and vCA1 (right) from representative ChR2 mice. Scale bars, 250 μm.

**c**, Behavior paradigm.

**d**, **e**, Stimulation of dCA3-originating axons in vCA1 induced social discrimination deficit. When receiving blue light stimulation, the ChR2 mice showed a significant decrease in time spent (**d**, n= 9 mice; OFF: P = 0.0002; ON: P = 0.8362; paired t test) interacting with and preference index (**e**, P = 0.0066, unpaired t test) for a stranger mouse, when compared to those before light stimulation.

**f**, The mice received optical stimulation in the test phase when performing the novel object recognition task.

**g**, **h**, The ChR2-expressed mice spent more time exploring the novel object than the familiar object with or without optical stimulation (**g**, n = 7 mice; OFF: P = 0.0044; ON: P = 0.0320; paired t test ). Optogenetic activation of dCA3-vCA1 pathway did not change the novel object preference index (**h**, P = 0.7807, unpaired t test).

**P < 0.01; ***P < 0.001; NS, not significant. Data presented as mean ± SEM.

**
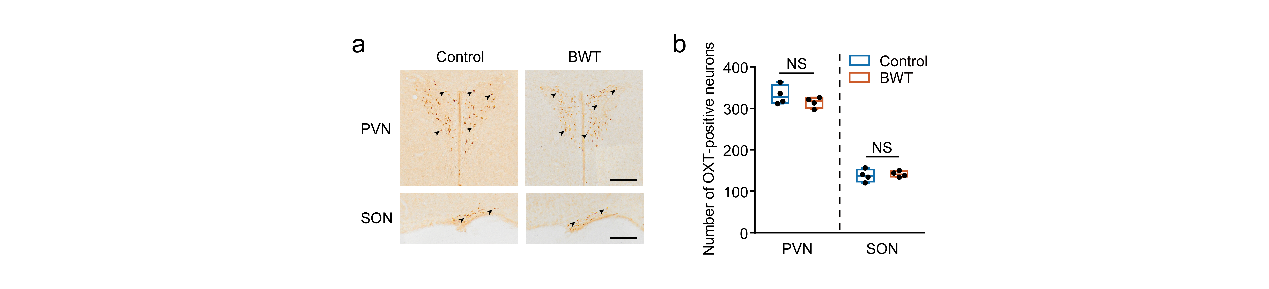
**

**Figure S10. The number of OXT-expressing neurons in the PVN and SON did not change in P14 mice undergoing bilateral whisker trimming at P12.**

**a**, Representative images of oxytocin-positive neurons in the PVN and SON at P14 in control and BWT mice. Black arrow indicates oxytocin posivitive cell. Scale bars, 200 μm.

**b**, Number of oxytocin-positive neurons in the PVN (n = 4 mice, each group; P = 0.4857) and SON (n = 4 mice, each group; P = 0.7429). Mann Whitney test.

NS, not significant. Error bars indicate SEM.

**
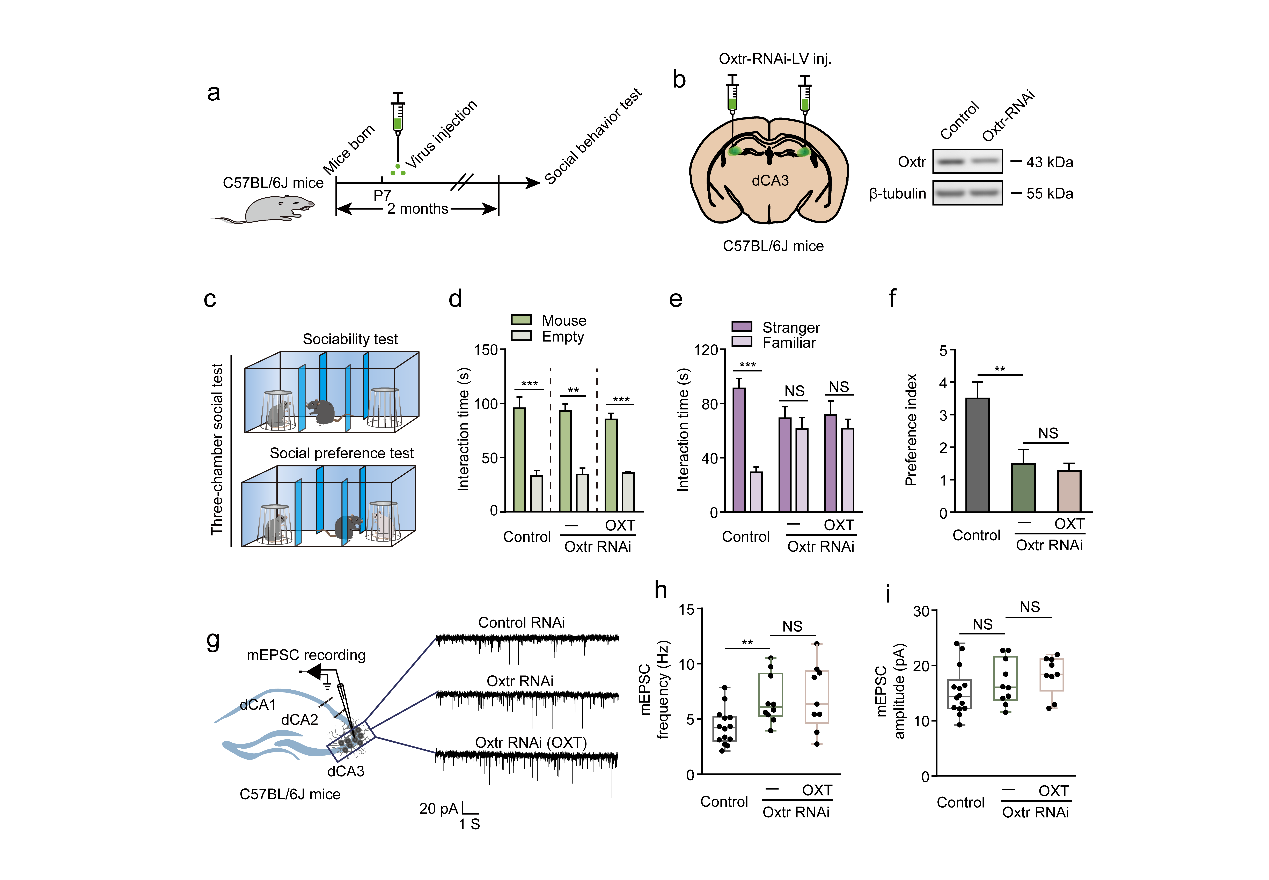
**

**Figure S11. Early life Oxtr knockdown impairs social behavior in adult and could not be rescued by concurrently neonatal OXT treatment.**

**a**, **b**, Behavioral schedule. P7 mice were bilaterally injected with Oxtr-RNAi-LV or control RNAi into the dCA3 and the social behavior test was performed two months later. Western blot analysis of oxytocin protein levels in dCA3 at P12 transfected with control or Oxtr RNAi (**b** right).

**c**-**f**, The three chamber test. **d**, Sociability test. Control: n = 7, P = 0.0007, paired t test; Oxtr RNAi: n = 8, P = 0.0078, Wilcoxon matched-pairs signed rank test; Oxtr RNAi (OXT): n = 6, P = 0.0005, paired t test. **e**, **f**, Social preference test. **e**, Control: P = 0.0008; Oxtr RNAi: P = 0.6323; Oxtr RNAi (OXT): P = 0.4629; paired t test. **f**, Control vs Oxtr RNAi: P = 0.0094; Oxtr RNAi vs Oxtr RNAi (OXT): P = 0.6949; unpaired t test.

**g**-**i**, mEPSC recording. **g**, Representative mEPSC recordings in dCA3 cells from control RNAi (upper), Oxtr RNAi and oxtr RNAi (OXT) groups (lower). **h**,

mEPSC frequencies. Control: 14 neurons from 3 mice; Oxtr RNAi: 10 neurons from 3 mice; Oxtr RNAi (OXT): 9 neurons from 4 mice. Control vs Oxtr RNAi: P = 0.3497, unpaired t test; Oxtr RNAi vs Oxtr RNAi (OXT): P = 0.4999, unpaired t test. **i**, mEPSC amplitudes. Control vs Oxtr RNAi: P = 0.0047, unpaired t test; Oxtr RNAi vs Oxtr RNAi (OXT): P = 0.8377, unpaired t test.

**P < 0.01; ***P < 0.001; NS, not significant. Data presented as mean ± SEM.

**
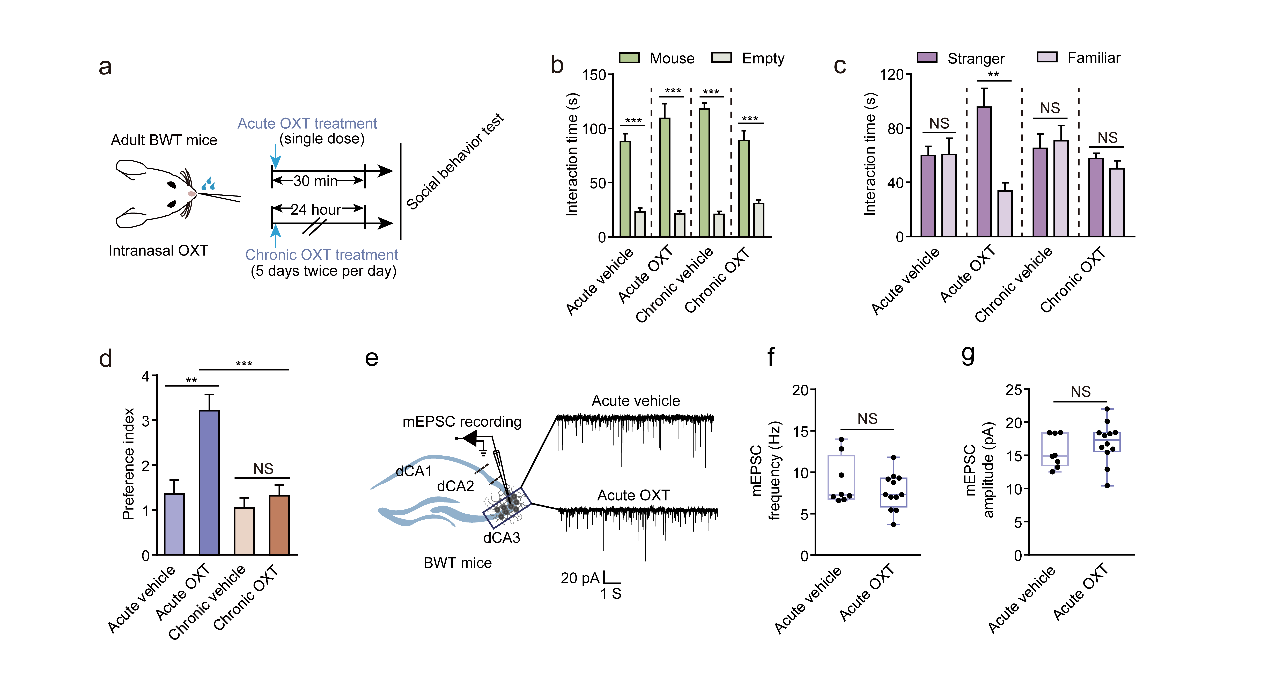
Figure S12. Effects of OXT treatment in adult BWT mice.**

**a**, Adult BWT mice received the acute or chronic intranasal OXT treatment before social behavior test.

**b**-**d**, Social behavior test. **b**, Time spent sniffing the mouse chamber or the empty chamber in sociability test (Acute vehicle: n = 9 mice, P = 0.0002; acute OXT: n = 10 mice, P = 0.0002; chronic vehicle: n = 8 mice, P < 0.0001; n = 6 mice; chronic OXT: n = 8 mice, P < 0.0001; paired t test). **c**, **d**, Time spent (**c**, Acute vehicle: P = 0.9544, paired t test; acute OXT: P = 0.0020, Wilcoxon matched-pairs signed rank test; chronic vehicle: P = 0.6251, paired t test ; chronic OXT: P = 0.3460, paired t test) and preference (**d**, Acute vehicle vs acute OXT: P = 0.0013; chronic vehicle vs chronic OXT: P = 0.4230; acute OXT vs chronic OXT: P = 0.0008; unpaired t test) for interacting with a stranger mouse versus a familiar mouse.

**e**-**g**, mEPSC recording. **e**, Representative spontaneous mEPSC recordings in dCA3 cells from acute vehicle (upper) and acute OXT groups (lower). **f**, **g**, mEPSC frequencies (f, P = 0.2840, unpaired t test) and mEPSC amplitudes (**g**, P = 0.3335, unpaired t test) in dCA3 (Acute vehicle, n = 8 neurons from 3 mice; OXT, n = 12 neurons from 3 mice).

**P < 0.01; ***P < 0.001; NS, not significant. Data presented as mean ± SEM.

**
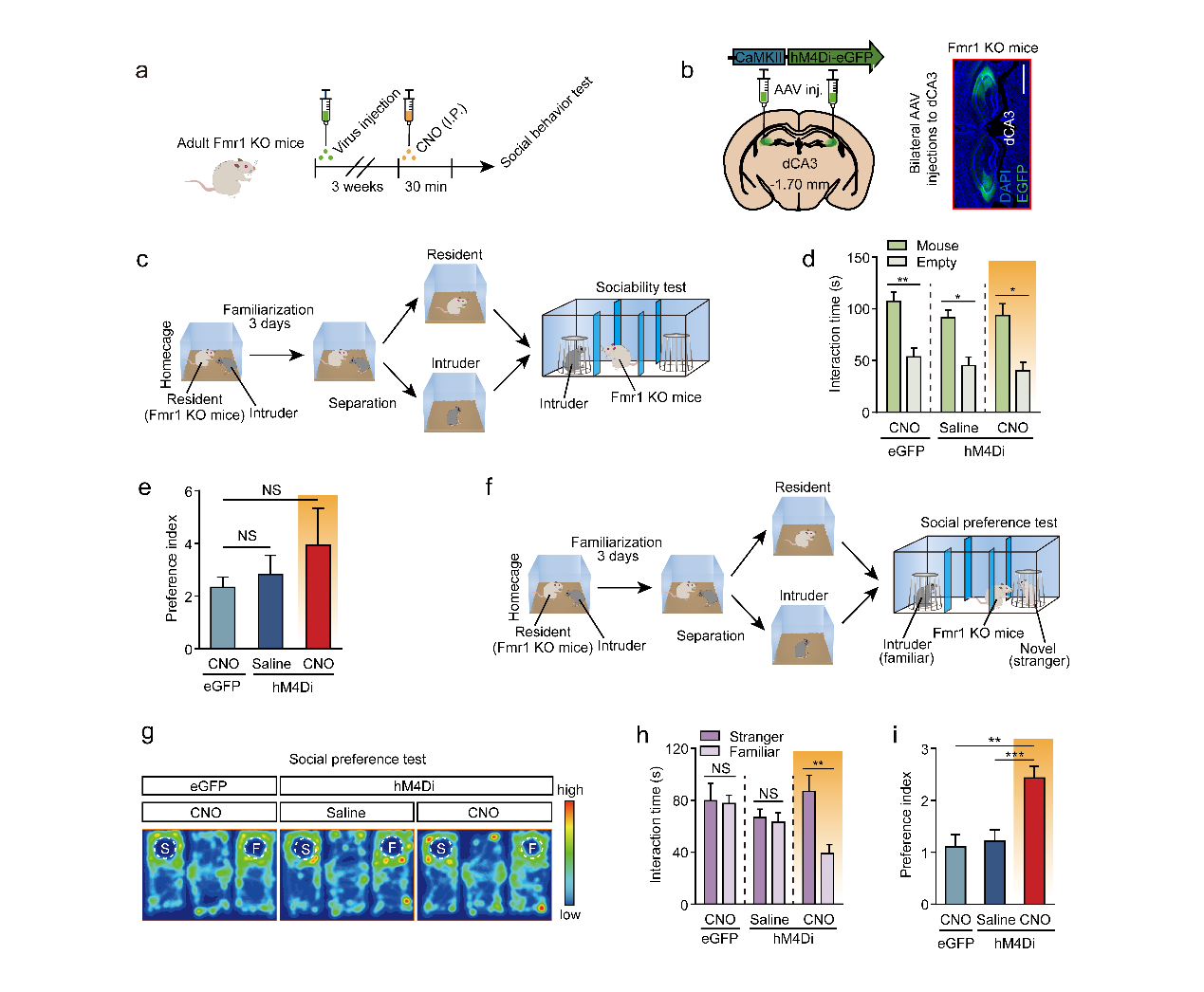
**

**Figure S13. Chemogenetic silencing of dCA3 rescues social discrimination deficits in Fmr1 KO mice.**

**a**, **b**, Adult Fmr1 KO mice were bilaterally injected with AAV-CaMKIIα-hM4Di-EGFP (or eGFP alone) into the dCA3 and a social behavior test was then performed. **b**, scale bar indicates 1 mm.

**c**-**e**, Sociability test for Fmr1 KO mice. Time spent (**d**) and preference (**e**) for sniffing the mouse chamber or the empty chamber. **d**, CNO (eGFP), n = 8 mice, P = 0.0016, paired t test; Saline(hM4Di), n = 8 mice, P = 0.0102, paired t test; CNO (hM4Di), n = 8 mice, P = 0.0134, paired t test. **e**, CNO (eGFP) vs CNO (hM4Di), P = 0.2871, unpaired t test; Saline (hM4Di) vs CNO (hM4Di), P = 0.4899, unpaired t test.

**f**, **i**, Social preference test. **g**, Representative heat maps from the social preference test. Time spent (**h**) and preference (**i**) for interacting with a stranger mouse versus a familiar mouse. **h**, CNO (eGFP), n = 8 mice, P = 0.8929; Saline (hM4Di), n = 8 mice, P = 0.6664,; CNO (hM4Di), n = 8 mice, P = 0.0002; Paired t test. **i**, CNO (eGFP) vs CNO(hM4Di), P = 0.0011; Saline (hM4Di) vs CNO (hM4Di), P = 0.0019; unpaired t test.

**P < 0.01; ***P < 0.001; NS, not significant. Data presented as mean ± SEM.

**
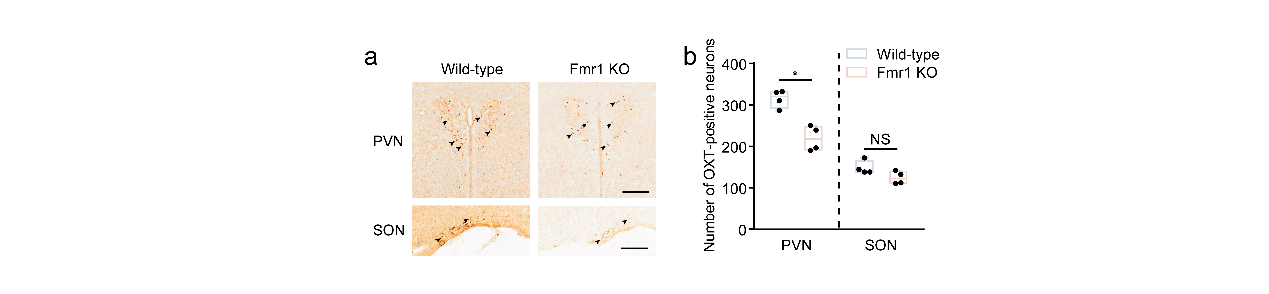
**

**Figure S14. Fmr1 KO mice show reduced number of OXT-expressing neurons in the PVN at P14.**

**a**, Representative images of OXT immunoreactivity in the PVN and SON at P14 in wild-type and Fmr1 KO mice. Black arrow indicates oxytocin posivitive cell. Scale bars, 200 μm.

**b**, Number of oxytocin-positive neurons in the PVN (n = 4 mice, each group; P = 0.0286) and SON (n = 4 mice, each group; P =0.1143). Mann Whitney test.

*P < 0.05; NS, not significant. Error bars indicate SEM.

**
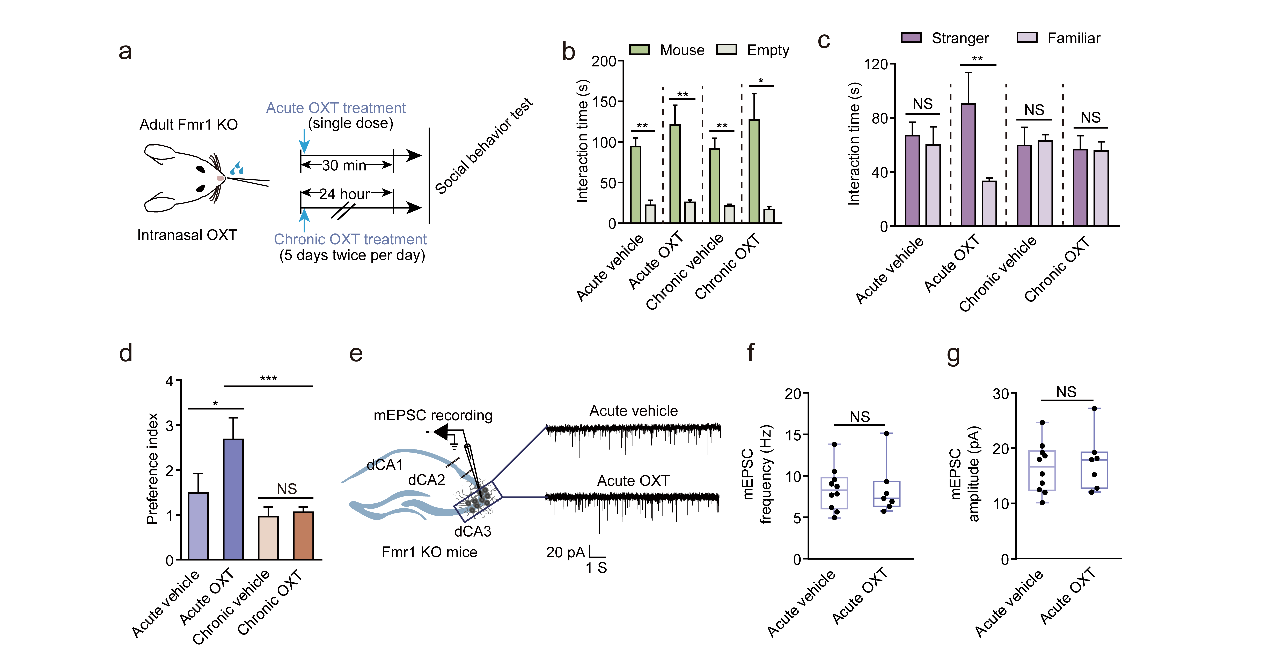
**

**Figure S15. Effects of OXT treatment in adult Fmr1 KO mice.**

**a**, Adult Fmr1 KO mice received the acute or chronic intranasal OXT treatment before social behavior test.

**b**-**e**, Social behavior test. **b**, Time spent sniffing the mouse chamber or the empty chamber in sociability test (Acute vehicle: n = 6 mice, P = 0.0011; acute OXT: n = 9 mice, P = 0.0044; chronic vehicle: n = 6 mice, P = 0.0032; n = 6 mice; chronic OXT: n = 6 mice, P = 0.0187; paired t test). **c**, **d**, Time spent (**c**, Acute vehicle: P = 0.7366, paired t test; acute OXT: P = 0.0039, Wilcoxon matched-pairs signed rank test; chronic vehicle: P = 0.8130, paired t test; chronic OXT: P = 8655; paired t test) and preference (**d**, Acute vehicle vs acute OXT: P = 0.0207, unpaired t test; chronic vehicle vs chronic OXT: P = 0.7922, unpaired t test; acute OXT vs chronic OXT: P = 0.0002, Mann-Whitney U test) for interacting with a stranger mouse versus a familiar mouse.

**e**-**g**, mEPSC recording. **e**, Representative spontaneous mEPSC recordings in dCA3 cells from acute vehicle (upper) and acute OXT groups (lower). **f**, **g**, mEPSC frequencies (**f**, P = 0.9843, unpaired t test) and mEPSC amplitudes (**g**, P = 0.6623, unpaired t test) in dCA3 (Acute vehicle, n = 10 neurons from 3 mice; Acute OXT, n = 7 neurons from 3 mice).

*P < 0.05; **P < 0.01; ***P < 0.001; NS, not significant. Data presented as mean ± SEM.

**
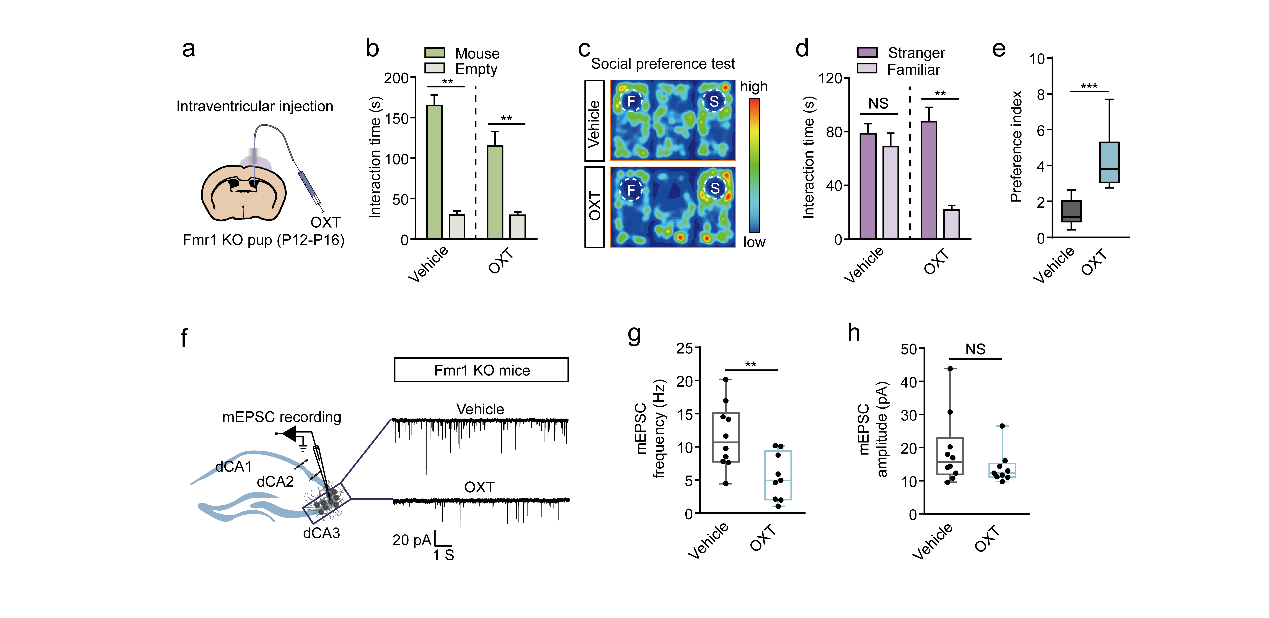
Figure S16. Social discrimination deficits in adult Fmr1 KO mice were rescued by early life intraventricular OXT treatment.**

**a**, Schematic shows intraventricular injection of OXT.

**b**, Time spent sniffing the mouse chamber or the empty chamber in sociability test (vehicle: n = 9 mice, P = 0.0039;. OXT: n = 11 mice, P = 0.0010; Wilcoxon matched-pairs signed rank test).

**c**-**e**, Social preference test. **c**, Representative heat maps. **d**, **e**, Time spent (**d**, Vehicle: n = 9 mice, P = 0.2369, paired t test. OXT: n = 11 mice, P = 0.0010, Wilcoxon matched-pairs signed rank test) and preference (**e**, P = 0.0008, Mann-Whitney U test) for interacting with a stranger mouse versus a familiar mouse.

**f**, Representative spontaneous mEPSC recordings in dCA3 cells from vehicle (upper) and OXT groups (lower).

**g**, **h**, mEPSC frequencies (**g**, P = 0.0306) and mEPSC amplitudes (**h**, P = 0.0304) in dCA3 (vehicle, n = 9 neurons from 3 mice; OXT, n = 8 neurons from 3 mice). unpaired t test.

**P < 0.01; ***P < 0.001; NS, not significant. Data presented as mean ± SEM.

**Supplementary Table 1**

**
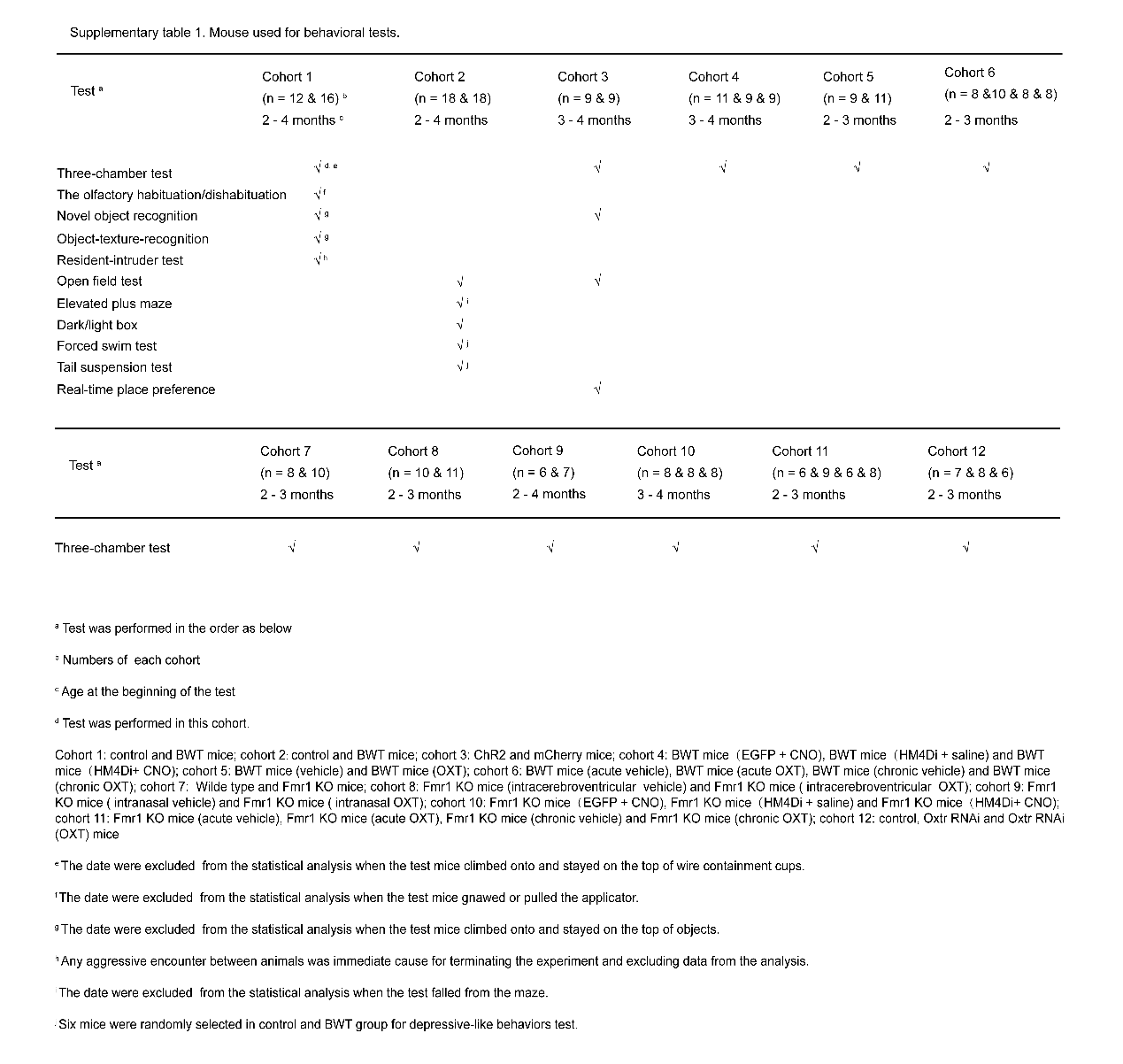
**

Information about the animal used in different behavioral tests.

**Supplementary Movie 1**

*In vivo* single-unit recording of the dorsal CA3 of a BWT mouse interacting with a familiar mouse during the social preference test.

**Supplementary Movie 2**

*In vivo* single-unit recording of the dorsal CA3 of a BWT mouse interacting with a strange mouse during the social preference test.

**Supplementary Movie 3**

A represented dorsal CA3 cell activity of a control mouse interacting with a familiar mouse during the social preference test.

**Supplementary Movie 4**

A represented dorsal CA3 cell activity of a control mouse interacting with a strange mouse during the social preference test.
